# Circulating levels of micronutrients and risk of osteomyelitis: a Mendelian randomization study

**DOI:** 10.3389/fnut.2024.1443539

**Published:** 2024-10-02

**Authors:** Xu Zhang, Jiaxing Wang, Zhimeng Wu, Binglong Xin, Shuixiang He, Zitong He, Yarui Li

**Affiliations:** ^1^Department of Gastroenterology, the First Affiliated Hospital of Xi’an Jiaotong University, Xi’an, China; ^2^Shannxi Clinical Research Center of Digestive Disease (Cancer Division), Xi’an, China; ^3^Health Science Center, Xi’an Jiaotong University, Xi’an, China; ^4^Shanxi Institute of Science and Technology, Jincheng, China

**Keywords:** micronutrients, two-sample Mendelian randomization, osteomyelitis, multivariable Mendelian randomization, infections

## Abstract

**Background:**

Few observational studies have investigated the effect of micronutrients on osteomyelitis, and these findings are limited by confounding and conflicting results. Therefore, we conducted Mendelian randomization (MR) analyses to evaluate the association between blood levels of eight micronutrients (copper, selenium, zinc, vitamin B12, vitamin C, and vitamin D, vitamin B6, vitamin E) and the risk of osteomyelitis.

**Methods:**

We performed the two-sample and multivariable Mendelian randomization (MVMR) to investigate causation, where instrument variables for the predictor (micronutrients) were derived from the summary data of micronutrients from independent cohorts of European ancestry. The outcome instrumental variables were used from the summary data of European-ancestry individuals (*n* = 486,484). The threshold of statistical significance was set at *p* < 0.00625.

**Results:**

We found a significant causal association that elevated zinc heightens the risk of developing osteomyelitis in European ancestry individuals OR = 1.23 [95% confidence interval (CI) [1.07, 1.43]; *p* = 4.26E-03]. Similarly, vitamin B6 showed a similar significant causal effect on osteomyelitis as a risk factor OR = 2.78 (95% CI [1.34, 5.76]; *p* = 6.04E-03; in the secondary analysis). *Post-hoc* analysis suggested this result (vitamin B6). However, the multivariable Mendelian randomization (MVMR) provides evidence against the causal association between zinc and osteomyelitis OR = 0.98(95% CI [−0.11, 0.07]; *p* = 7.20E-1). After searching in PhenoScanner, no SNP with confounding factors was found in the analysis of vitamin B6. There was no evidence of a reverse causal impact of osteomyelitis on zinc and vitamin B6.

**Conclusion:**

This study supported a strong causal association between vitamin B6 and osteomyelitis while reporting a dubious causal association between zinc and osteomyelitis.

## 1 Introduction

Osteomyelitis is an inflammatory process accompanied by bone destruction and caused by infections (1), which is a serious complication in orthopedic surgery. With reported infection rates of orthopedic trauma patients ranging from 5 to 10% (2, 3), orthopedic infections are associated with significantly increased patient morbidity (4, 5) and medical costs (6, 7). Multiple micronutrients have been shown to have important roles in the immune system. They are essential components of immune cell proliferation, maturation, cytokine release, and enzymes involved in immune cell activity for antioxidant host defense. Those deficiencies significantly impair host immunity and increase susceptibility to infection (8).

Observational studies have reported different results about the effect of vitamin D on osteoarticular infections. Signori et al. found that a high level of vitamin D was one of the negative prognostic factors for prosthetic joint infections (9). By contrast, Marschall et al. proposed that there was no association between vitamin D deficiency and negative clinical outcomes in osteoarticular infections (10). However, evidence from these observational studies was insufficient to demonstrate a causal relationship and was limited by not only sample selection bias but also confounding environmental factors. Apart from them, few studies reported the association between other micronutrients and osteomyelitis.

Mendelian randomization (MR) represents a novel form of evidence synthesis and causal inference that is becoming increasingly significant in epidemiological research (11). A significant benefit of this methodology, particularly pertinent to nutritional epidemiology, is the capacity to accurately ascertain genotypes and discern long-term exposure patterns (12). MR enables causal inference using genetic variants as proxies for risk factors and health outcomes susceptible to confounding factors (13). MR uses available genetic data with single nucleotide polymorphisms (SNPs) that are correlated with exposures (in this case micronutrients) as instrumental variables to evaluate the causal relationship between the exposure and the outcome of interest (in this case osteomyelitis) (14, 15). It can limit problems of reverse causation and unknown confounding factors typical of conventional observational epidemiology (15).

Multivariable Mendelian randomization (MVMR) represents a recent extension to Mendelian randomization (MR) that employs genetic variants associated with multiple, potentially related exposures to estimate the effect of each exposure on a single outcome. This approach preserves the advantages of employing genetic instruments for causal inference, such as circumventing bias due to confounding while allowing for the inclusion of multiple exposures in the model (16, 17). This enables the estimation and moderation of the direct causal relationship between each exposure and the outcome (18, 19).

However, MR is susceptible to some important limitations. These include the lack of high-quality data, violations of three basic assumptions, limited biological understanding of gene-exposure associations, trait heterogeneity, and so on (12).

Since the relationship between micronutrients and osteomyelitis has not been explored by any genetic instruments, we hypothesized there was a causative association of osteomyelitis with micronutrients. Therefore, we set out to use two-sample and MVMR methods to estimate the causal relationship between genetically predicted blood levels of micronutrients and the genetically predicted risk of osteomyelitis. We identified eight micronutrients of interest that have been associated with the risk of infection and for which there are available genetic instruments—copper, selenium, zinc, vitamin B12, vitamin C, vitamin B6, vitamin E, and vitamin D—and assessed the risk of osteomyelitis.

## 2 Methods

### 2.1 Study design

**Figure 1:**
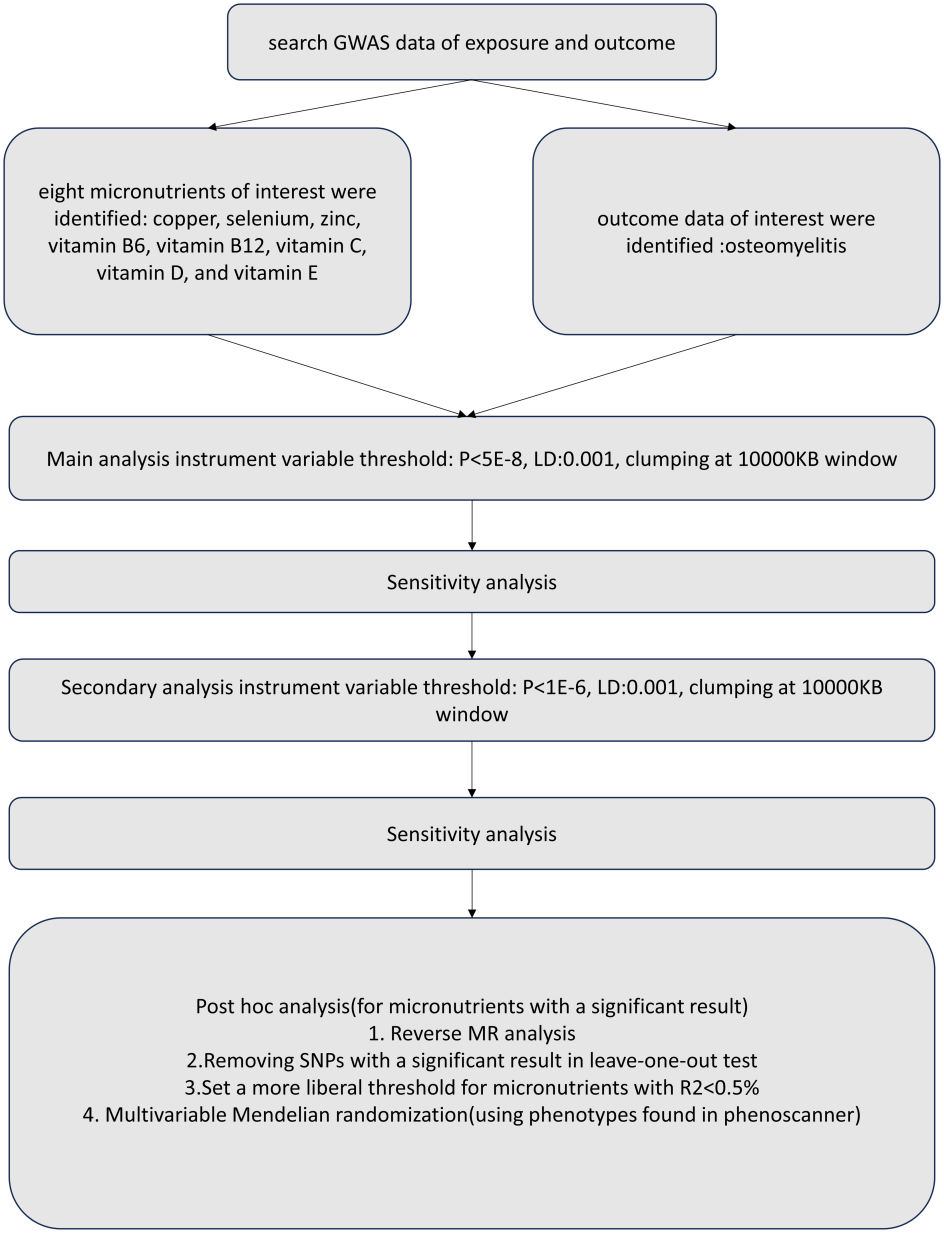
A schematic summary of the study design.

We conducted a two-sample MR and MVMR study using publicly available summary statistics. To reduce bias from population stratification (20), both the exposure and outcome cohorts were restricted to subjects of European ancestry. Figure 1 provides a schematic summary of the study design.

### 2.2 GWAS data sources

We selected genetic instruments from 8 publicly available GWAS summary statistics of all individuals of European ancestry as exposures from the GWAS catalog, IEU OpenGWAS, and PubMed, including copper (21), selenium (21), zinc (21), vitamin B6, vitamin B12 (22), vitamin C (23), vitamin D (24), and vitamin E (details showed in Supplementary Table S1). Genetic instruments for osteomyelitis were obtained from a summary statistic based on 4,836 cases of osteomyelitis and 481,648 controls (25), all of whom were of European ancestry. More relevant information is shown in Supplementary Table S9 and Table 1. Osteomyelitis cases were defined by M00, M01, M46, and M86 in ICD-10 codes, and controls were defined by the absence of the above ICD codes.

**Table 1:** Source of exposure genome-wide association study summary data.

Exposure	Main analysis		Secondary analysis			Population ancestry	Data source
	Number of SNPs	% of variance explained		Number of SNPs	% of variance explained		
Cu	2	4.60		2	4.60	European	(21)
Se	1	1.93	QIMR	3	3.86	European	(21)
	1	2.89	ALSPAC	3	4.78	European	
Zn	2	4.59		5	7.57	European	(21)
Vitamin B6	1	0.05		4	0.17	European	Ieu: ukb-b-7864
Vitamin B12	6	1.85		9	2.23	European	(22)
Vitamin C	11	1.78		17	2.09	European	(23)
Vitamin D	105	2.70		164	2.93	European	(24)
Vitamin E	0	–		6	0.23	European	Ieu: ukb-b-6888

### 2.3 Two-sample Mendelian randomization

After genetic instrument selection and harmonization, the inverse-weighted variance (IVW) method was used to perform the two-sample MR analysis. In the absence of exposure-outcome heterogeneity and directional pleiotropy, this method has been reported to provide reasonably accurate estimates (26). We use the MR-Egger regression to check for the potential existence of horizontal pleiotropy between instrumental variables (27). The MR-Egger intercept value deviates significantly from zero with a *p* < 0.05 as the evidence of horizontal pleiotropy (26, 28). In the first *post-hoc* analysis for the micronutrients with significant associations, we removed the SNPs with *p* < 0.05 in the leave-one-out test. For the explanation of variance<0.5%, we adopted a wider limit of threshold (*p* < 1E-05, LD < 0.001, clump at a 10,000 KB window) in the secondary *post-hoc* analysis.

### 2.4 Multivariable Mendelian randomization

The Multivariable Mendelian Randomization method can distinguish the role of multiple phenotypes associated with SNPs in causality association (18). Therefore, for the micronutrients with a significant result in the two-sample MR, we searched phenotypes associated with SNPs on PhenoScanner and applied MVMR to verify whether any of the identified phenotypes may interfere with the effect of micronutrients on osteomyelitis. We included three phenotypes in the MVMR analysis of zinc: mean corpuscular volume (GWAS identifier: ukb-d-30040), mean corpuscular hemoglobin concentration (GWAS identifier: ukb-d-30060), reticulocyte count (GWAS identifier: ukb-d-30250) after searching on PhenoScanner. And no phenotype related to vitamin B6 was found.

### 2.5 Sensitivity analyses

For the selection of SNPs, to avoid bias, only IVs that met the three assumptions of MR were used: the relevance assumption, exclusion restriction assumption and the independence assumption. The first assumption is that the SNPs used as instrumental variables should be closely associated with exposure factors. The second assumption is that the candidate instrumental variables should not be associated with confounding factors. The third assumption is that the proposed genetic variants should only influence the risk of the health outcome via the exposure we have focused on. For the first assumption, all the instrumental variables applied in the main analyses were significantly correlated with the exposure for the two-sample MR at *p* < 5E-8. In the secondary analyses, we use variants at a more liberal threshold *p* < 1E-06. Furthermore, we validated the second MR assumption by selecting only IVs with L.D < 0.001 after clumping in a 10,000 kb window. These instruments that met the first two MR assumptions were then subjected to the downstream sensitivity analyses.

We also used the systematic leave-one-out method to detect potential pleiotropy per SNP to test compliance with the exclusion restriction assumption for the micronutrients containing >2 SNPs. The robust penalized IVW estimate was used to assess the causality effect. Then we evaluated the change before and after the removal of each SNP. Heterogeneity between instrumental variables was tested using Q-statistics with *p* < 0.05. We evaluate our instrument strength using the proportion of variance interpreted (R2) and the F-statistic (29, 30). Only instrument variables with *F* ≥ 10 can be selected to perform MR. We further checked the adherence to the exclusion restriction assumption of MR by using the MR-Egger regression through its intercept terms.

### 2.6 Statistical analysis

Statistical analysis was performed using the inverse-variance weighted (IVW) method to estimate the causal effects for instruments that satisfied the instrumental variable assumptions (13). To exclude the false causal effect due to horizontal pleiotropy, we conducted an MVMR including traits searched on PhenoScanner using instrumental variables involved in Two-sample MR at *p* < 5E-08. We further tested for reverse causality by performing an MR analysis considering osteomyelitis as exposure and micronutrients as an outcome. Two MR analyses were conducted in this study (main analysis and secondary analysis). The main analyses were applied using all the instrumental variables selected as those significantly correlated with the exposure at *p* < 5E-8. In the secondary analyses, we used exposure instrumental variables at a more liberal threshold *p* ≤ 1E-06. Statistical significance for causal relationships was set at *p* < 0.05. In multiple tests, the *p*-value adjusted by Bonferroni correction to *p* < 0.05/8 = 0.00625 was believed statistically significant. All analyses were conducted using TwoSampleMR and Mendelian Randomization R package in Rstudio (version 2023.9.1.494).^1^ A schematic summary of the study design was provided in Figure 1.

## 3 Results

**Figure 2:**
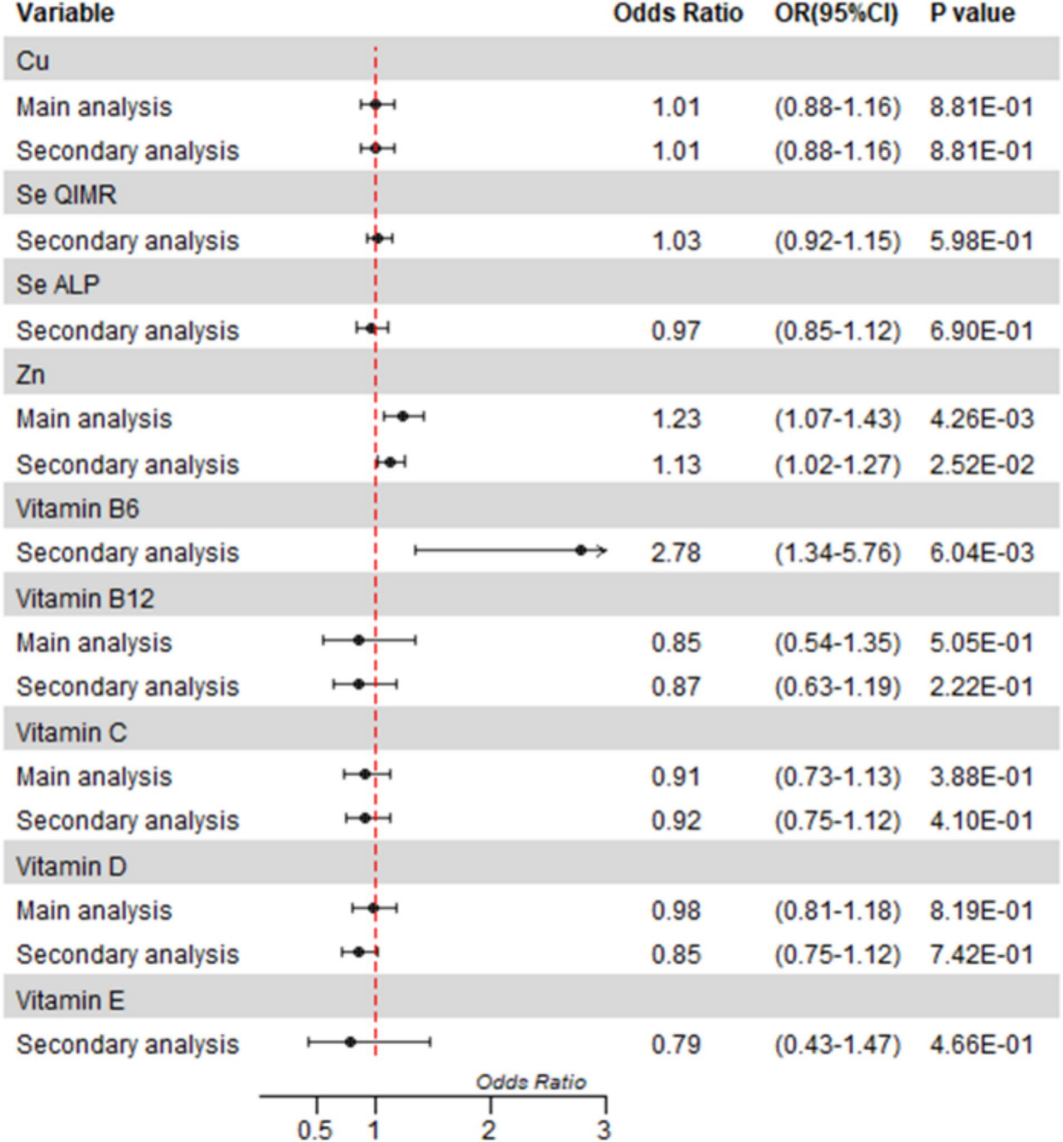
Mendelian randomization analyses of circulating micronutrient levels on osteomyelitis risk. Forest plot of inverse-variance weighted Mendelian randomization analyses. The x-axis represents the results expressed per standard deviation increase in genetically proxied levels of exposure. Cu, copper; Fe, Zn, zinc; Se, selenium.

The MR analysis was conducted as shown in Figure 2. Further details can be found in Supplementary materials.

### 3.1 Associations between micronutrients and osteomyelitis

After correction for multiple tests, we only found a statistically significant causal association between blood levels of zinc and osteomyelitis in the main analysis (OR = 1.23, 95%CI = 1.07 to 1.43; *p* = 0.0043). We observed little evidence that the blood levels of Cu, vitamin B12, vitamin C, and vitamin D were associated with the risk of osteomyelitis in the main analysis (Figure 2, details shown in the Supplementary materials). Because fewer than two SNPs were selected, analyses of selenium vitamin B6 and vitamin E were inconclusive.

In the secondary analysis, we found a nominally significant causal association between zinc and osteomyelitis (OR = 1.13, 95%CI = 1.02 to 1.27; *p* = 0.0252) and a strong causal association between vitamin B6 and osteomyelitis in the secondary analysis (OR = 2.78, OR 95%CI = 1.34 to 5.76; *p* = 0.0060). There was no significant association between other micronutrients and osteomyelitis in the secondary analysis.

### 3.2 Sensitivity analysis

Only data with more than two instrument variables selected were conducted horizontal pleiotropy tests and heterogeneity tests using MR-Egger. Additionally, we performed leave-one-out analyses for micronutrients containing >2 SNPs. Because only 2 significant instruments were selected for the main analysis of zinc, the horizontal pleiotropy test was not conducted. For zinc, the heterogeneity test was conducted by IVW and MR-Egger in the secondary analysis and IVW in the main analysis, and no evidence of heterogeneity in the instruments. In the secondary analysis, we found no evidence of horizontal pleiotropy between IVs using the MR-Egger regression intercept analysis. We further performed a leave-one-out analysis to estimate any horizontal pleiotropy and found all instrument variables with *p* < 0.05 except rs1532423 and rs2120019 (5 SNPs in total). We tested for weak instruments by calculating the F-statistics and all instruments with *F* > 10, and we found no evidence of horizontal pleiotropy and heterogeneity for the reverse analysis.

In the analyses of vitamin B6, we only perform sensitivity analysis for the secondary analysis because of no MR result for the main analysis. In the secondary analysis, we found no evidence of horizontal pleiotropy using the MR-Egger regression intercept analysis, and heterogeneity using IVW and MR-Egger between IVs. We further performed a leave-one-out analysis to estimate any horizontal pleiotropy and found rs155599 and rs3772928 with a *p* < 0.05 (4 SNPs in total). We tested for weak instruments by calculating the F-statistics and all instruments with *F* > 10. For the reverse analysis, we found no evidence of horizontal pleiotropy and heterogeneity (details shown in the Supplementary materials).

In the main and secondary analyses, Cu, Se, vitamin D, and vitamin C showed no evidence of horizontal pleiotropy and heterogeneity. Vitamin B12 showed a significant heterogeneity test result in the main and secondary analyses. We did not conduct a sensitivity analysis for vitamin E in the main analysis, because no SNP was selected in the main analysis for vitamin E. No evidence of horizontal pleiotropy and heterogeneity was observed in the secondary analysis of vitamin E. We test the strength of instrument variables using an F-statistic and all the instrument variables selected with an *F* > 10. Leave-one-out analysis results were displayed in the Supplementary materials.

### 3.3 *Post-hoc* analysis

There was no evidence of reverse causality between osteomyelitis and zinc or vitamin B6, as displayed in Supplementary Table S8. The MVMR analysis showed no statistically significant causal associations for genetically predicted zinc on osteomyelitis (OR = 0.98, 95%CI = 0.90 to 1.08, *p* = 0.7204). For zinc, we removed all the SNPs with a *p* < 0.05 in the leave-one-out test and found the SNPs left were the same as the SNPs contained in the main analysis. Therefore, the result of *post-hoc* analyses was also the same as the main analysis.

For vitamin B6, considering the leave-one-out analysis, we removed two SNPs (rs155599 and rs3772928) from the SNPs and performed a two-sample MR in the first *post-hoc* analysis. However, the analysis was limited by an insufficient number of SNPs included, leading to an undesirably low proportion of variance explained and an excessively wide 95%CI for OR. Then we conducted a two-sample MR using a more liberal threshold with removal of the SNPs with a *p* < 0.05 in the leave-one-out analysis for the secondary *post-hoc* analysis. The first *post-hoc* analysis of vitamin B6 showed a strong causal effect between vitamin B6 and osteomyelitis (OR = 5.92, OR 95%CI = 1.98 to 17.69; *p* = 0.0015). And the secondary *post-hoc* analysis of vitamin B6 reported a consistent result (OR = 2.22, 95%CI = 1.54 to 3.20; *p* = 0.00002) with the first *post-hoc* analysis.

## 4 Discussion

In this European-ancestry MR study, we investigated the causal associations of genetically predicted micronutrients on osteomyelitis using a two-sample and multivariable MR method. We found evidence of a significant causal association between blood levels of zinc and vitamin B6 in this European population. The results of zinc and vitamin B6 exceeded our predictions. Additionally, the reverse MR-IVW analysis showed a non-significant causal effect between osteomyelitis and either of the genetically predicted zinc and vitamin B6. However, there are parts of these results that do not support a causal relationship between osteomyelitis and zinc and need to be discussed in more detail.

We conducted both univariable and MVMR for the effect of the blood levels of zinc on osteomyelitis and showed contradictory results. In the main analysis, we found a significant association. However, in the secondary analysis, the result did not reach our adjusted threshold (*p* = 0.0252). After the removal of all SNPs with a *p* < 0.05 in the leave-one-out analysis, the results were the same as the main analysis. It indicated that the non-significant results of the secondary analysis were mainly influenced by these SNPs.

The result showed no evidence of the association between zinc and osteomyelitis after conditioning in the MVMR analysis. Noteworthy, due to limitations in data quality, only one SNP (rs12544332) strongly associated with zinc was included in the MVMR lower than the covariates we included in the MVMR. Thus, we calculated the F-statistic of zinc in MVMR, and the result was just 1.33, much lower than 10. We suspected it was a false negative result (16, 17). However, as no significant results were obtained in the second analysis or MVMR, this conclusion should be treated with more caution.

Zinc plays a key role in the development and function of the immune system (31, 32). A study showed that zinc supplementation could decrease the incidence of infections in the elderly (33), which contradicts our findings. Interestingly, zinc was also found to play a vital role in the bacteria (34). Another study found virtually no detectable zinc in tissue abscesses caused by *Staphylococcus aureus*, in contrast to the high levels of zinc found in the surrounding healthy tissue (35). *Staphylococcus aureus* is the main pathogen responsible for osteomyelitis (1). Due to the importance of zinc in the bacteria (34), vertebrates sequestering zinc from these abscesses could be seen as a nutritional immunity (36) strategy exploiting *Staphylococcus aureus*’s requirement for zinc to control infection. Although the mechanisms and function are not yet clear, it is evident that shortages in available zinc possibly disrupt several bacterial processes that are crucial to infection (37). This may indicate that a high blood level of zinc might damage the zinc sequestration by the host and promote the development of osteomyelitis. However, the available evidence does not provide sufficient support to make a robust conclusion that a high blood level of zinc is a risk factor for osteomyelitis. More powerful studies between zinc supplementation and osteomyelitis should be conducted.

For the post-hoc analysis of vitamin B6, we removed two questionable SNPs with *p* < 0.05 using leave-one-out analysis, thus avoiding the two SNPs influencing the analysis result and giving a false positive result. Furthermore, considering the low explanation of variance and too large OR 95%CI, we removed the SNPs with a *p* < 0.05 in the leave-one-out analysis and contained move SNPs at a *p*<1e-5 to report a more robust result (38). In the secondary analysis, vitamin B6 exhibited borderline statistical significance (OR = 2.78, 95%CI = 1.34 to 5.76; *p* = 0.0060), which potentially undermines the robustness of our conclusion. However, we performed the *post-hoc* analysis and found a significant result (OR = 5.92, OR 95%CI = 1.98 to 17.69; *p* = 0.0015) when we removed two SNPs with *p* < 0.05 in the leave-one-out analysis. Furthermore, we perform another *post-hoc* analysis with a wider threshold (SNPs with *p* < 1E-5, removal of SNPs with *p* < 0.05 in the leave-one-out analysis). It reported a result further away from the borderline (OR = 2.22, 95%CI = 1.54 to 3.20; *p* = 0.00002). Two *post-hoc* analyses reached the same conclusion and were far from the borderline, which enhanced the credibility of our conclusion. And the sensitive analyses of them showed negative results. The absence of an impact from the strict threshold on the outcome suggests that the result is relatively robust. The result showed vitamin B6 may be a potential risk factor for osteomyelitis. The reverse analysis ruled out the reverse causal association. Previous studies have suggested that vitamin B6 may have a protective effect against inflammation (39, 40). However, several studies challenged this conclusion. Davis et al. reported that controlled dietary vitamin B6 restriction did not correlate with the CRP levels. Another study showed that in people with stable angina pectoris, levels of inflammatory markers did not improve after supplying high-dose (40 mg/d) PN alone or in combination with folic acid and vitamin B12 (41). Notably, multiple studies have shown that plasma serine and glycine (with an anti-inflammatory effect) were increased after a period of vitamin B6 deficiency (42–44) due to reduced glycine decarboxylase (GDC, a PLP-dependent enzyme) (44). This may indicate that a high level of vitamin B6 plays a negative effect on the accumulation of glycine and its anti-inflammatory effects contributing to osteomyelitis.

Pyridoxal 5’-phosphate (PLP) is a major present form of vitamin B6 *in vivo* and plays an important role as a co-factor in the KYNU enzyme in the tryptophan pathway (45). The increase of KYNU produced significant direct inflammatory effects, and its tryptophan pathway downstream metabolites had significant direct inflammatory effects on a variety of cells such as keratinocytes, T cells, and ECs. Subsequent studies have confirmed that KYNU expression is significantly higher than IDO and TDO in a variety of inflammatory diseases (46). Increased PLP promoted an increase in the downstream products catalyzed by KYNU, which may be the reason why vitamin B6 induced osteomyelitis in our results.

Steven et.al found the rates of homocysteine remethylation or synthesis remained unaltered in a dietary vitamin B6 restriction (< 0.5 mg/d) for 4 weeks (42). This may be considered as a reference for a vitamin B6 restriction diet in the clinical trials of osteomyelitis. It is imperative to underscore the glaring absence of sufficient clinical trials that directly demonstrate the effects of dietary vitamin B6 restriction on individuals suffering from osteomyelitis. Therefore, dietary vitamin B6 restriction should be approached with the utmost prudence. Detailed clinical suggestions still need more clinical trials of dietary vitamin B6 restriction or supplements in the future.

Therefore, we further suggested future studies to conduct more powerful experiments to find the direct association and mechanism between vitamin B6 and osteomyelitis.

We found no relationship between genetically predicted circulating Cu, Se, vitamin D, vitamin B12, vitamin E, and vitamin C and the risk of osteomyelitis. Systematic reviews of RCTs have found limited evidence of the association between vitamin C, Se supplementation, and infections but have also highlighted the limitations of studies (47, 48). Padhani ZA et al. found low-quality evidence for vitamin C supplementation in the protection of pneumonia (47). The other review also found no strong evidence in favor of selenium supplementation for the development of infections and the incidence of new infections (48) in critically ill patients. Another retrospective cohort study showed that baseline hypovitaminosis D is not associated with poor clinical outcomes in osteoarticular infections (10). A similar Mendelian-Randomization study found an association between blood level of copper and gastrointestinal infections but had no evidence of the effect of vitamin B12 on gastrointestinal infections, pneumonia, and urinary tract infections. However, only two SNPs were contained for copper in their study (49). We did not find any other robust studies supporting the association between copper and osteomyelitis. A double-blind, placebo-controlled trial found the effect of one-year supplementation with 200 IU/day vitamin E is a protective factor for respiratory infections (50). However, no evidence of an association between vitamin E and osteomyelitis was found.

Notably, although diagnostic criteria were consistent in the process of collecting osteomyelitis data, it still had biases for the differences in diagnosis criteria among different regions, centers, and people. It may affect the accuracy of our results. Furthermore, the utilization of single-time-point blood levels as proxies for long-term exposure may introduce biases, we suggested using GWAS data derived from multiple blood samples or multiple biological samples in future studies.

Our study strengths were in the use of continental European-derived GWAS summary statistics to assess the causal association between 8 micronutrients (copper, selenium, zinc, vitamin B6, vitamin B12, vitamin C, vitamin D, and vitamin E) and osteomyelitis and conduct further analyses for zinc and vitamin B6 including reverse MR, MVMR, and *post-hoc* analysis. Sensitivity analyses were also performed, including multi-variable MR-Egger and F-statistic, to verify the reliability of our instrumental variables, as detailed in the methods section.

### 4.1 Limitations

The study was limited by a lack of available separate GWAS data for vitamin B12, vitamin C, and vitamin D to decrease the risk of selection bias and the bias due to overlapping samples. The sample size of our study cohorts was insufficient to yield a more robust result. Furthermore, the weak instrument bias affects our MVMR result. We only included 2 SNPs for zinc and 1 SNPs for vitamin B6 in the primary analysis. Thus, we conducted secondary analysis and *post-hoc* analysis using more lenient *p*-value thresholds to include more SNPs. However, the lower p threshold applied in the secondary analysis and *post-hoc* analysis may introduce bias. Some demographic characteristics, environmental factors, or comorbidities have been considered in the original GWAS research. We regretted to fail to obtain the detailed data and consider them in our study. However, MR uses available genetic data with SNPs that are correlated with exposures (in this case micronutrients) as instrumental variables to evaluate the causal relationship between the exposure and the outcome of interest (in this case osteomyelitis). It may reduce the impact of environmental factors.

To minimize the risk of population stratification, we only contained European ancestry participants. However, this study still could be potential population stratification. And inclusion of only European populations affects the external validity of our findings to other ancestry groups. Analyses of micronutrients in other ethnic groups should be performed to increase the validation of generalizations.

Due to the unavailability of high-quality data, the analyses of other micronutrients with close associations with bone metabolism and immune regulation were excluded for further investigation. This may fail to identify potential causal links. The intention was to collect data about other micronutrients and to undertake a study examining the relationship between other micronutrients and osteomyelitis.

We regret that additional health economics analyses and cell and animal experiments were not conducted as part of this study. Nevertheless, we have decided to conduct a series of health economics analyses and functional experiments to further investigate micronutrients and osteomyelitis.

## 5 Conclusion

Our study reported a significant result of the causal association between vitamin B6 and osteomyelitis and a dubious causal association between zinc and osteomyelitis. Furthermore, we found that there was no evidence of any link between Cu, Se, vitamin D, vitamin B12, vitamin E, and vitamin C and osteomyelitis. Nevertheless, there are some limitations in this study. More studies were required to substantiate our conclusions and to learn more about the potential underlying mechanisms. Our findings have several important implications. Firstly, the questionable link between zinc and osteomyelitis suggests that the nutritional immunology theory may be a key factor in the development of osteomyelitis. This required us to focus our attention on the mechanisms of zinc in the development of osteomyelitis. Second, we have uncovered that vitamin B6 may potentially emerge as a risk factor for osteomyelitis, a find that contradicts previous studies and suggests the need for reevaluating vitamin B6 supplementation strategies in the context of infectious diseases, potentially paving the way for a novel approach. Third, this study supports no association between Cu, Se, vitamin D, vitamin B12, vitamin E, and vitamin C and osteomyelitis, which may reduce unnecessary vitamin supplementation and lower healthcare costs.

## Data Availability

The datasets presented in this study can be found in online repositories. The names of the repository/repositories and accession number(s) can be found in the article/Supplementary material.

## Footnotes

^1^ www.rstudio.com
