# Supplementary material for: Circulating levels of micronutrients and risk of osteomyelitis: a Mendelian randomization study
Source: Front Nutr. 2024 Oct 2;11:1443539. doi: 10.3389/fnut.2024.1443539 (PMC11479910; doi:10.3389/fnut.2024.1443539)
Supplement: Supplementary file 1 [file Data_Sheet_1.docx]

Supplementary Material

# Supplementary Figures and Tables

## Supplementary Figures


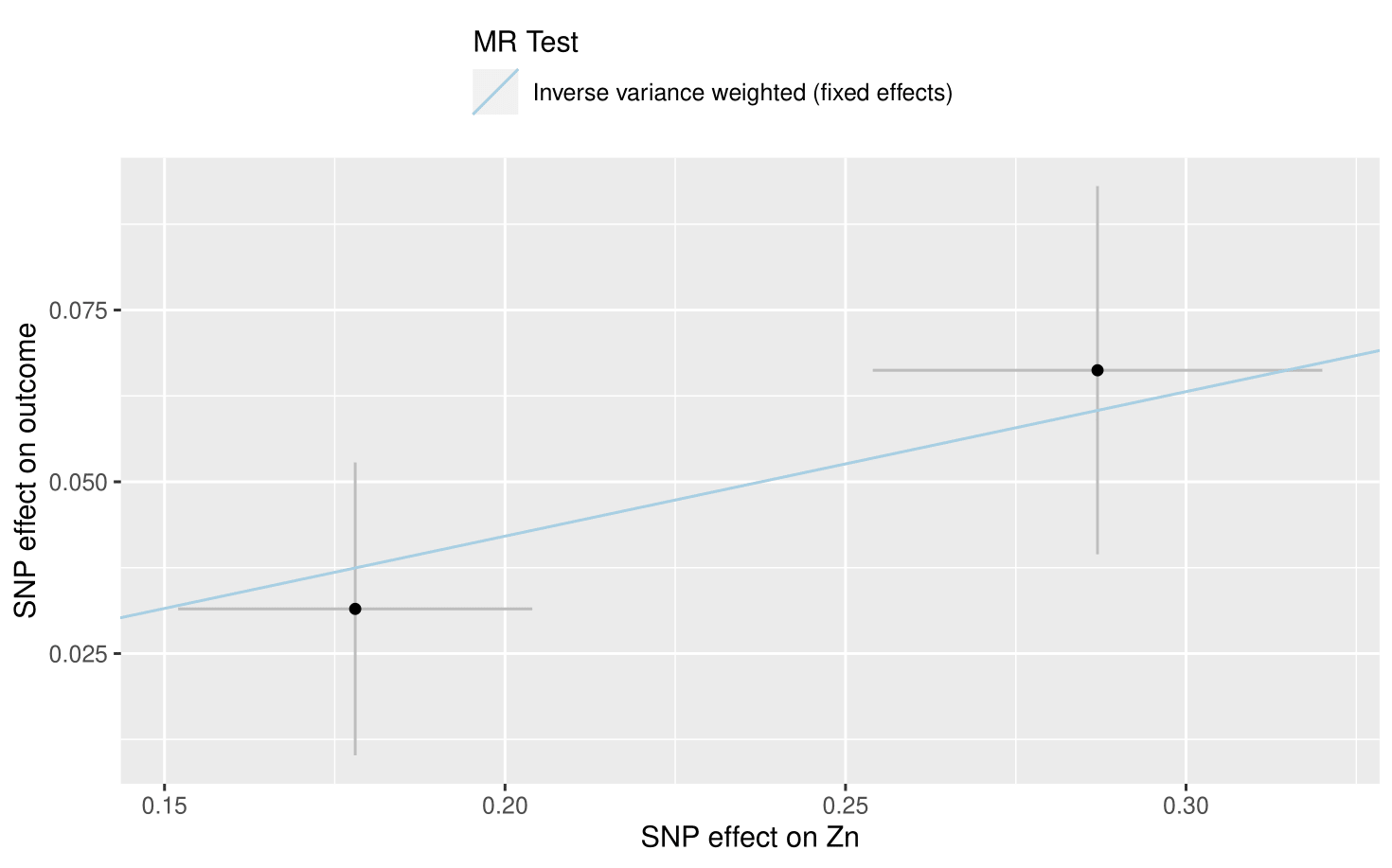


**Fig.S1**:Scatter plot of main analysis of zinc as risk factors on the risk of osteomyelitis


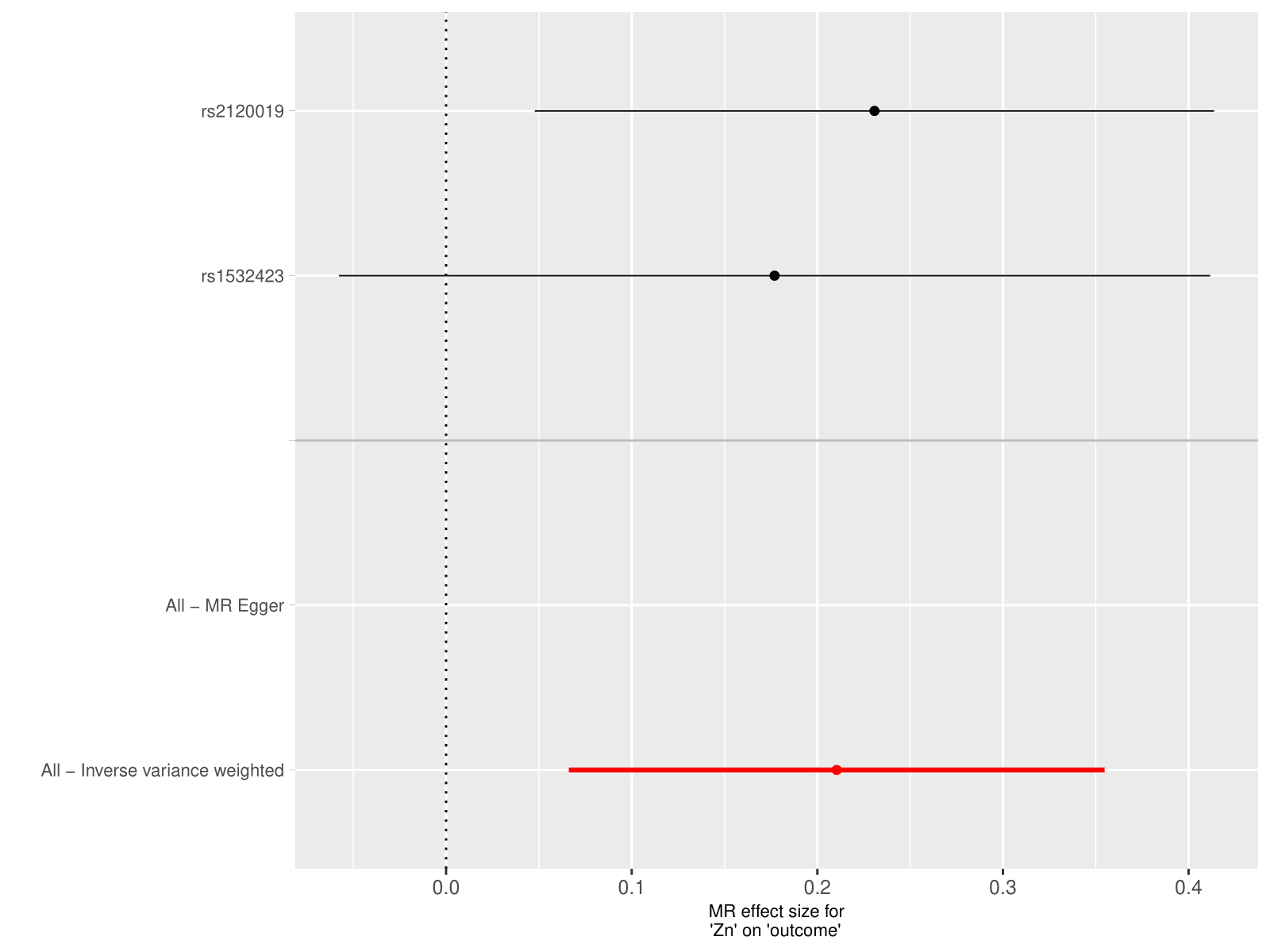


**Fig.S2**: Forest plot of main analysis of zinc as risk factors on the risk of osteomyelitis


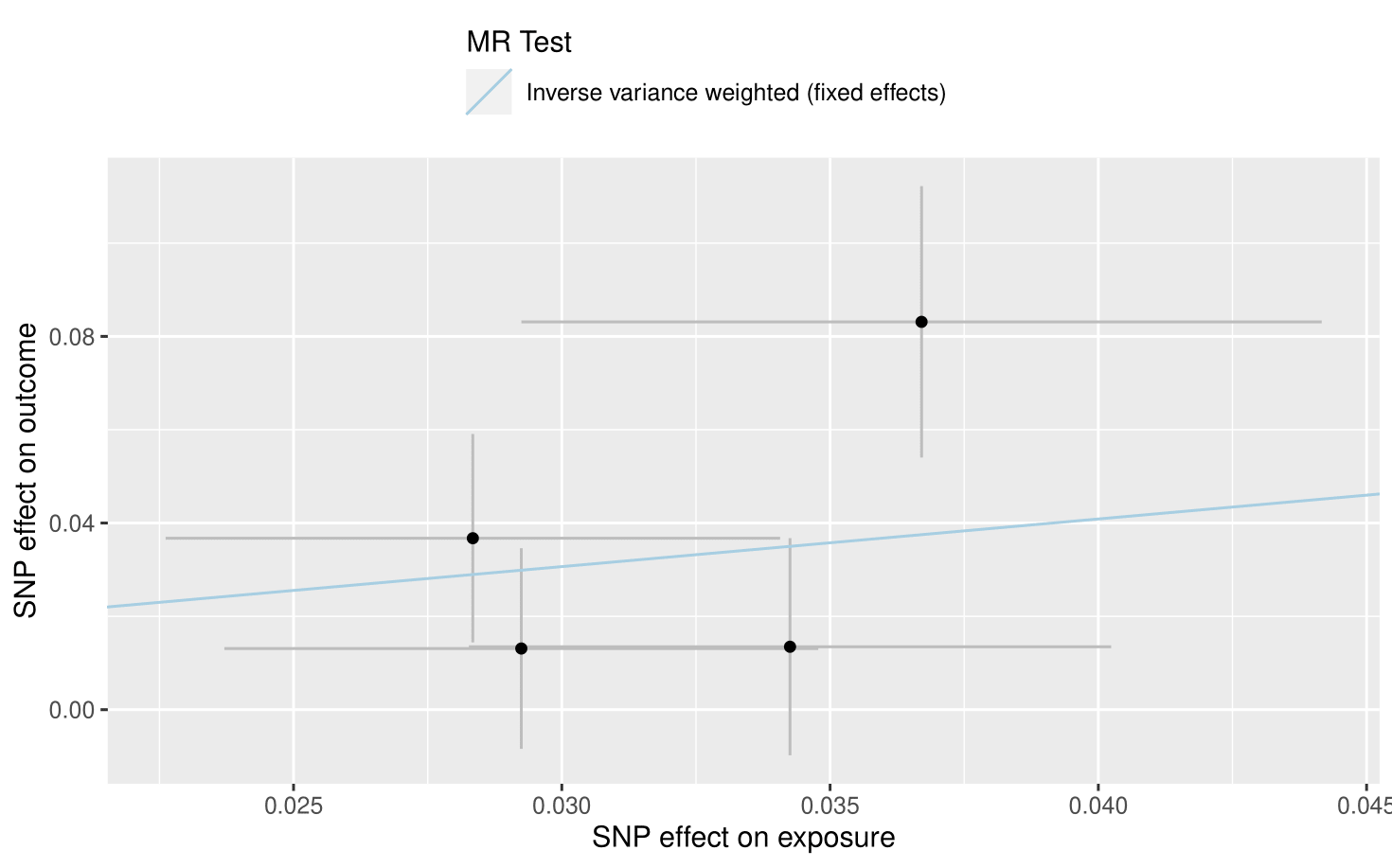


**Fig.S3**: Scatter plot of secondary MR analysis of vitamin B6 as risk factors on the risk of osteomyelitis

**
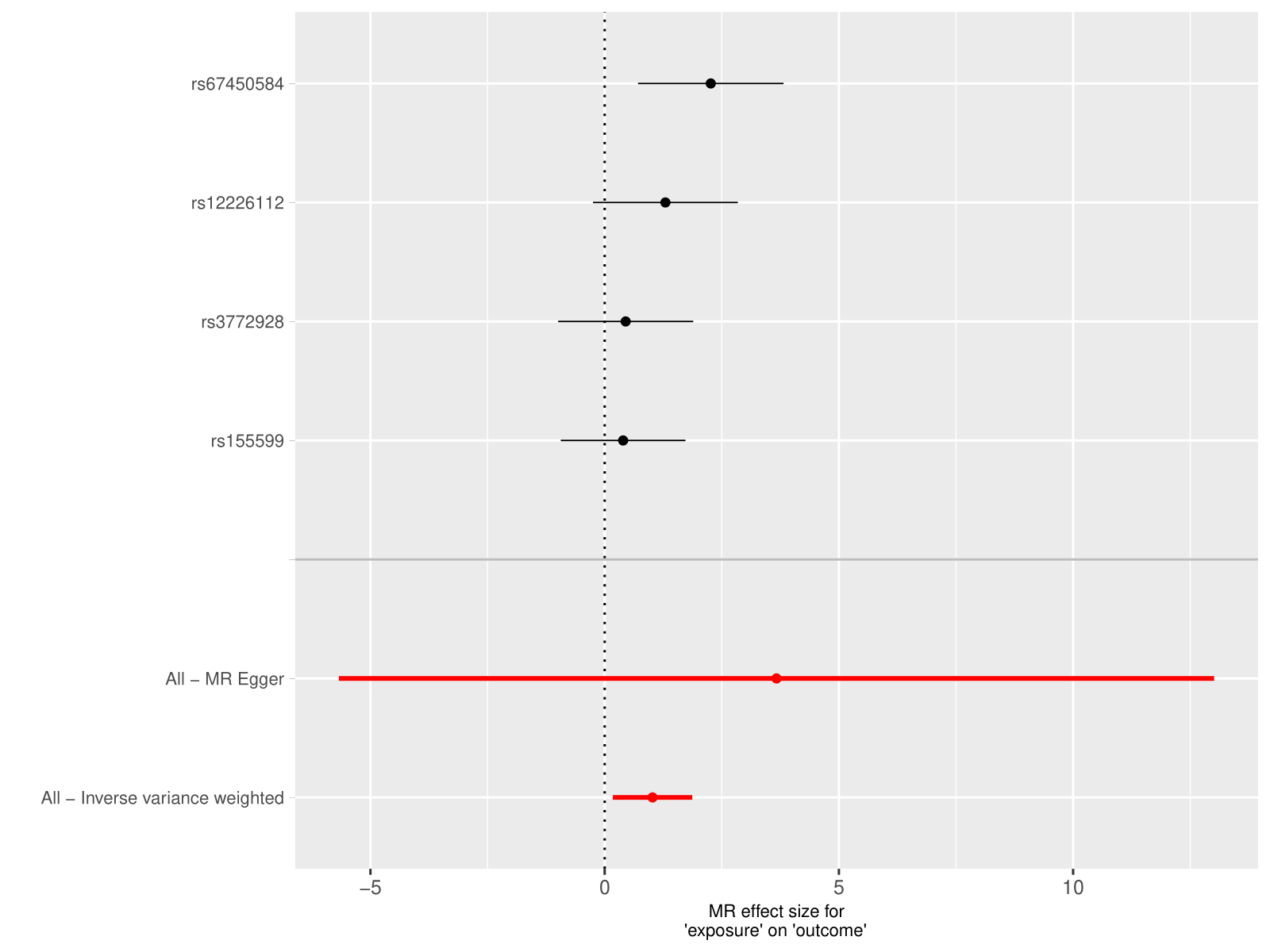
**

**Fig.S4**: Forest plot of secondary MR analysis of vitamin B6 as risk factors on the risk of osteomyelitis

**
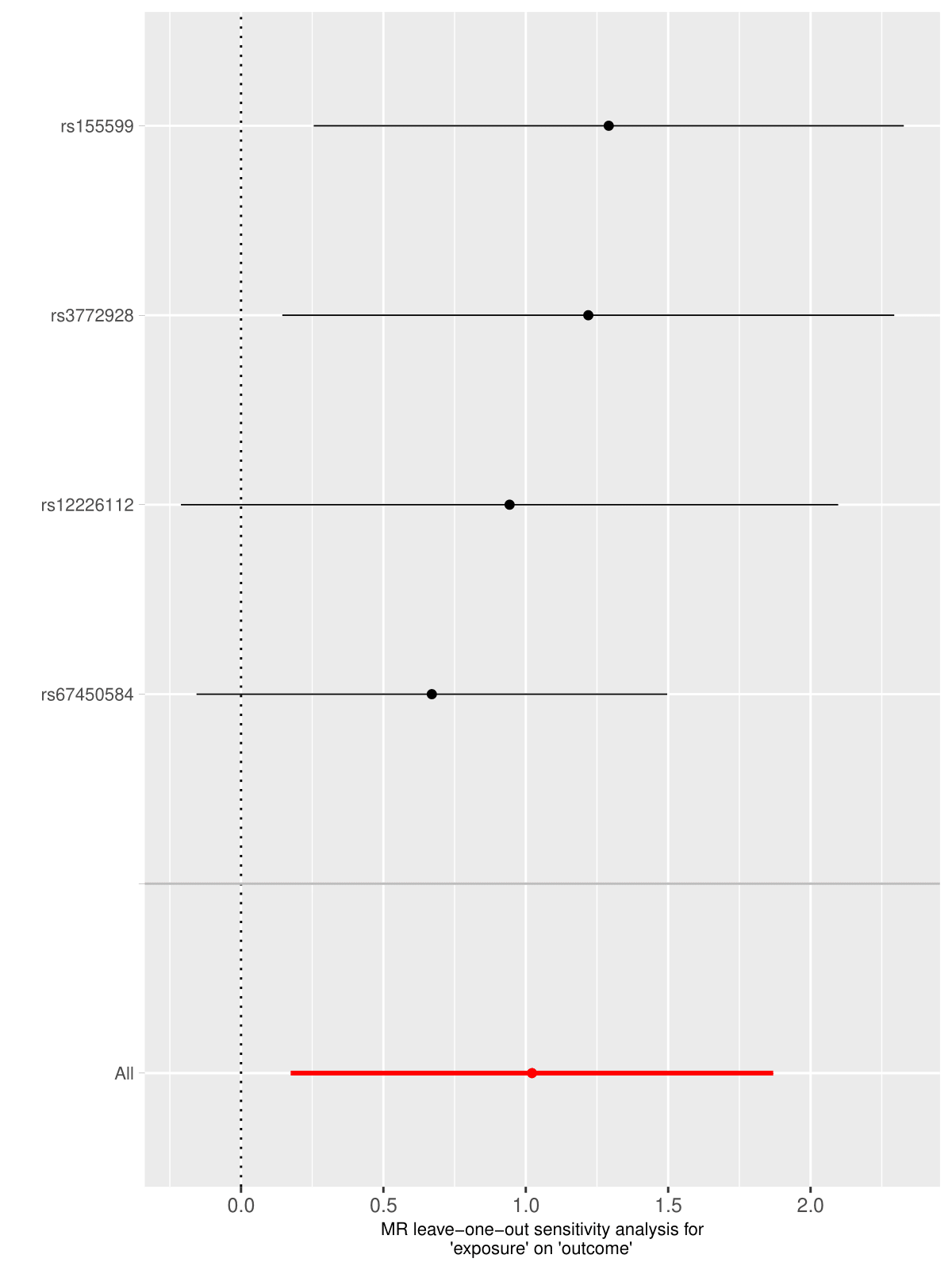
**

**Fig.S5**: Leave-one-out plot of secondary MR analysis of vitamin B6 as risk factors on the risk of osteomyelitis

**
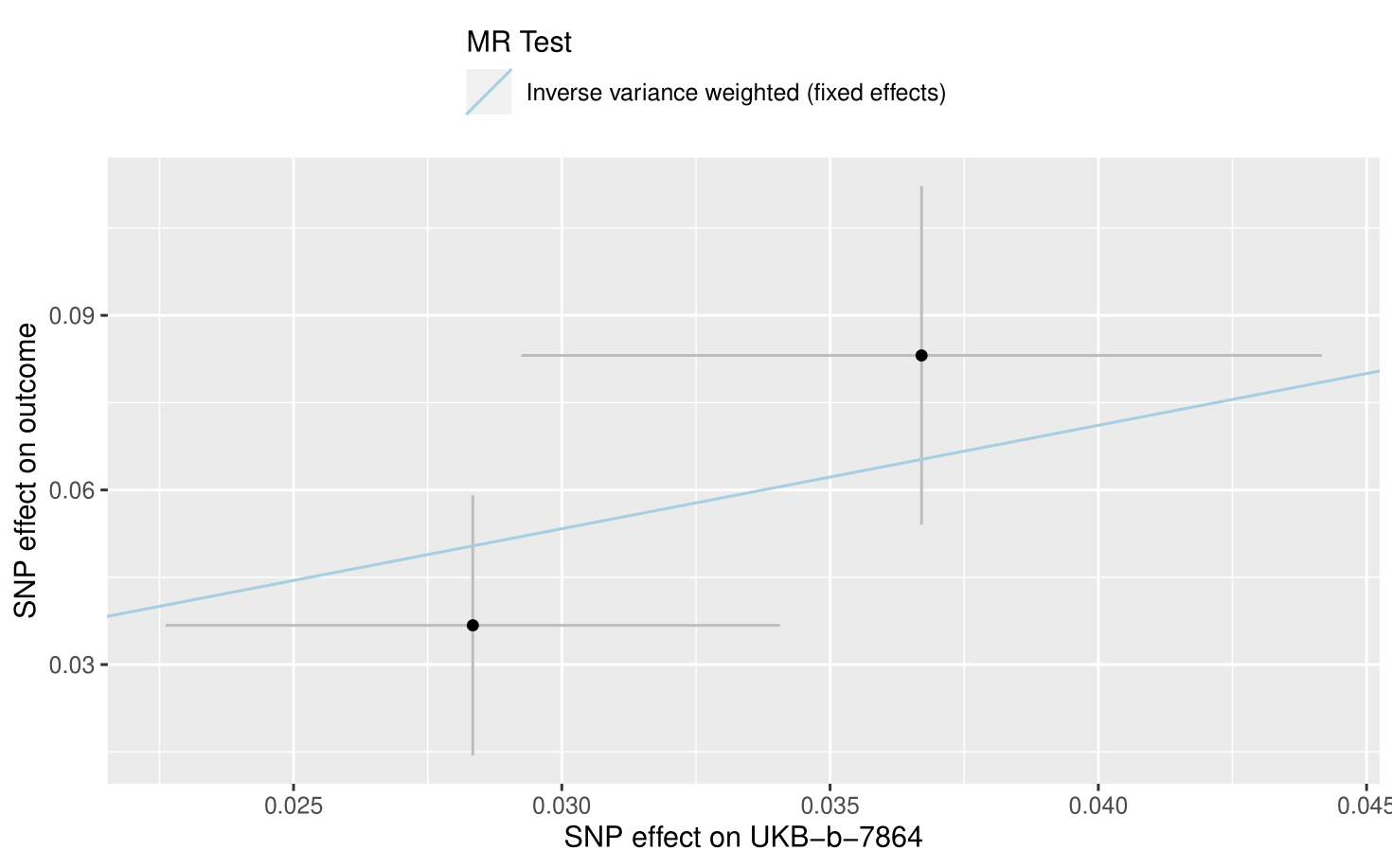
**

**Fig.S6**: Scatter plot of first post hoc analysis of vitamin B6 as risk factors on the risk of osteomyelitis (r2 < 0.001 within 10,000 kb windows and P ≤ 1E-06 and removing SNPs with a p<0.05 in the leave-one-out analysis)

**
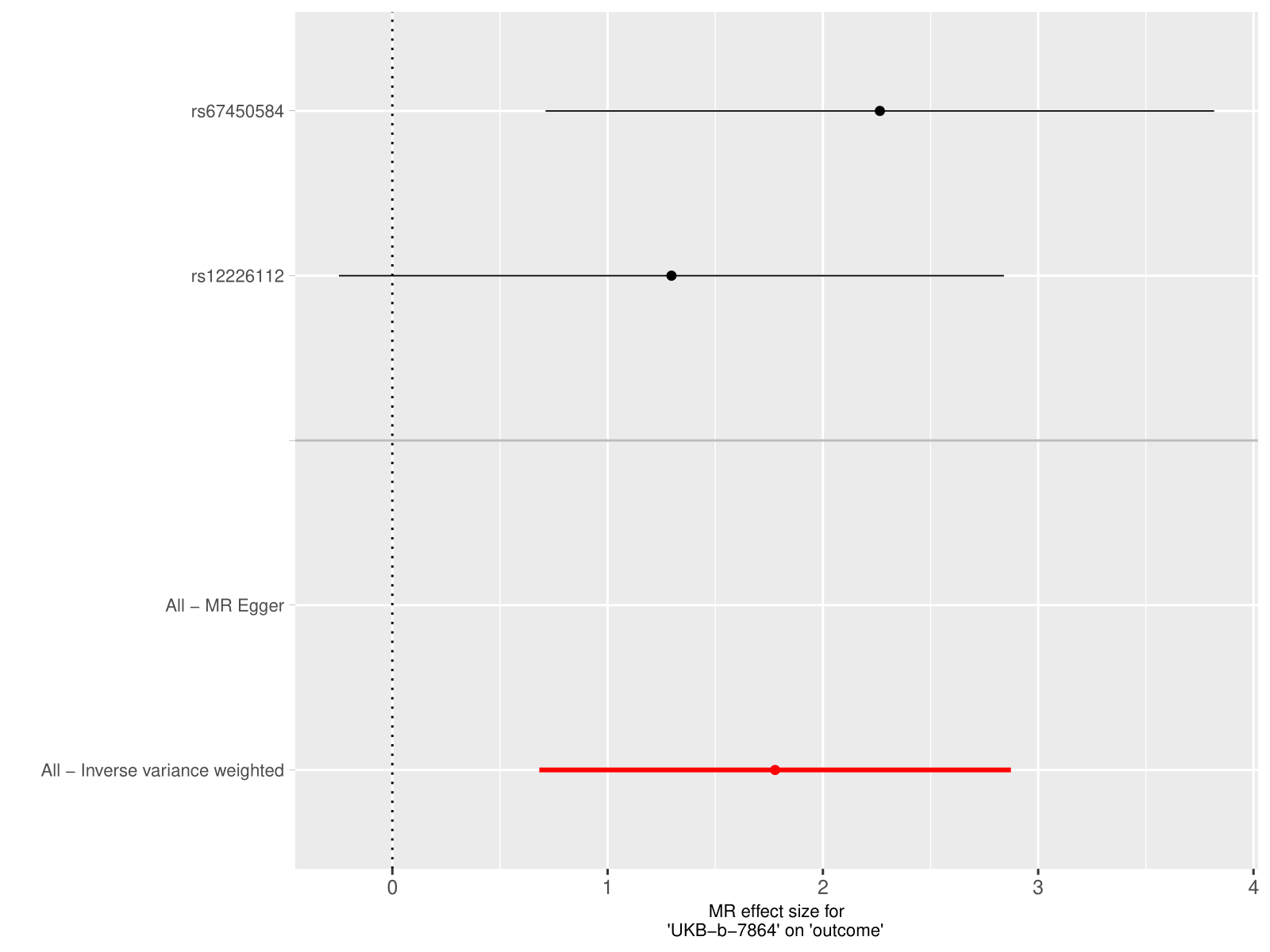
**

**Fig.S7**: Forest plot of first post hoc analysis of vitamin B6 as risk factors on the risk of osteomyelitis (r2 < 0.001 within 10,000 kb windows and P ≤ 1E-06 and removing SNPs with a p<0.05 in the leave-one-out analysis)

**
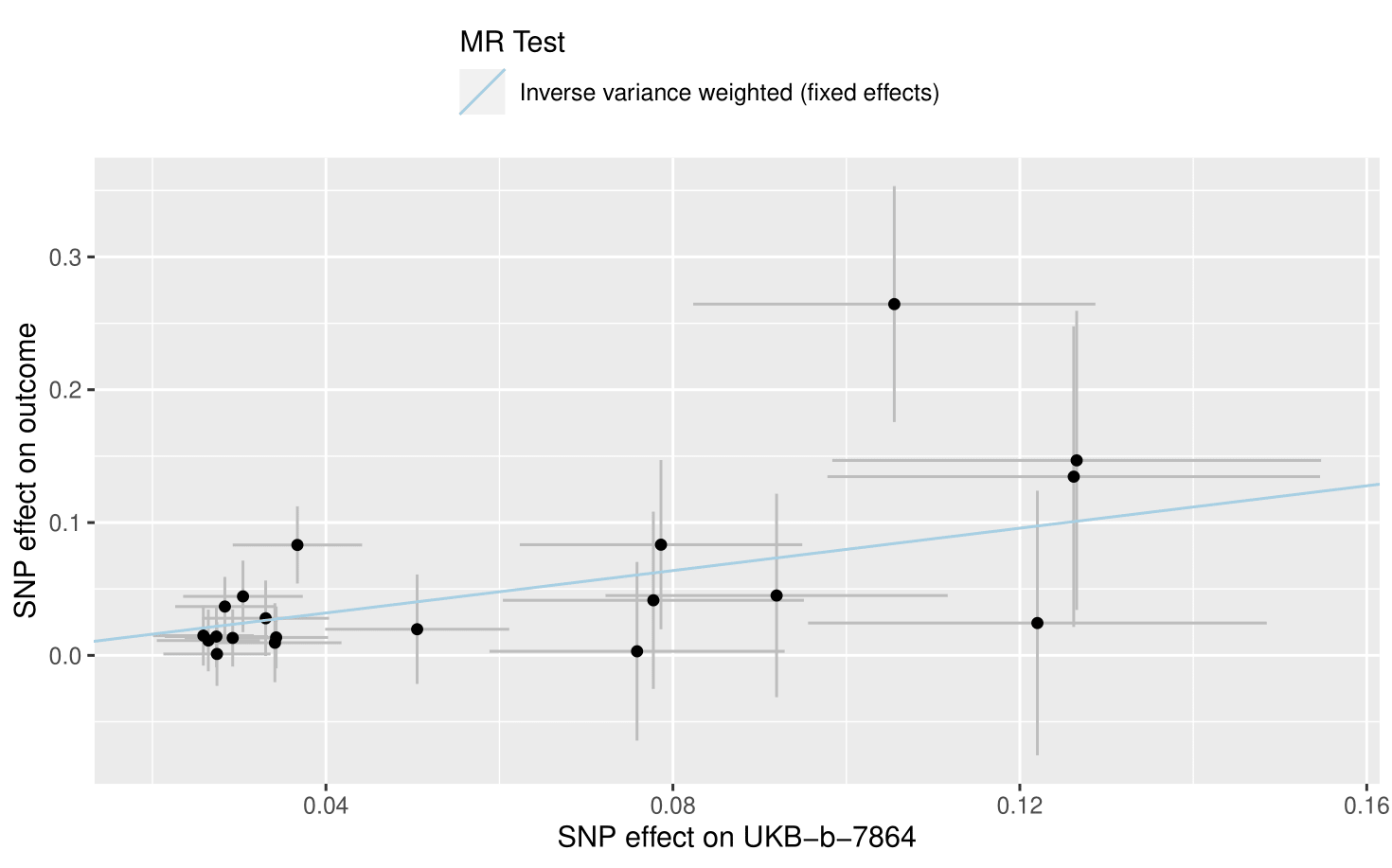
**

**Fig.S8**: Scatter plot of secondary post hoc analysis of vitamin B6 as risk factors on the risk of osteomyelitis (r2 < 0.001 within 10,000 kb windows and P ≤ 1E-05 and removing SNPs with a p<0.05 in the leave-one-out analysis)

**
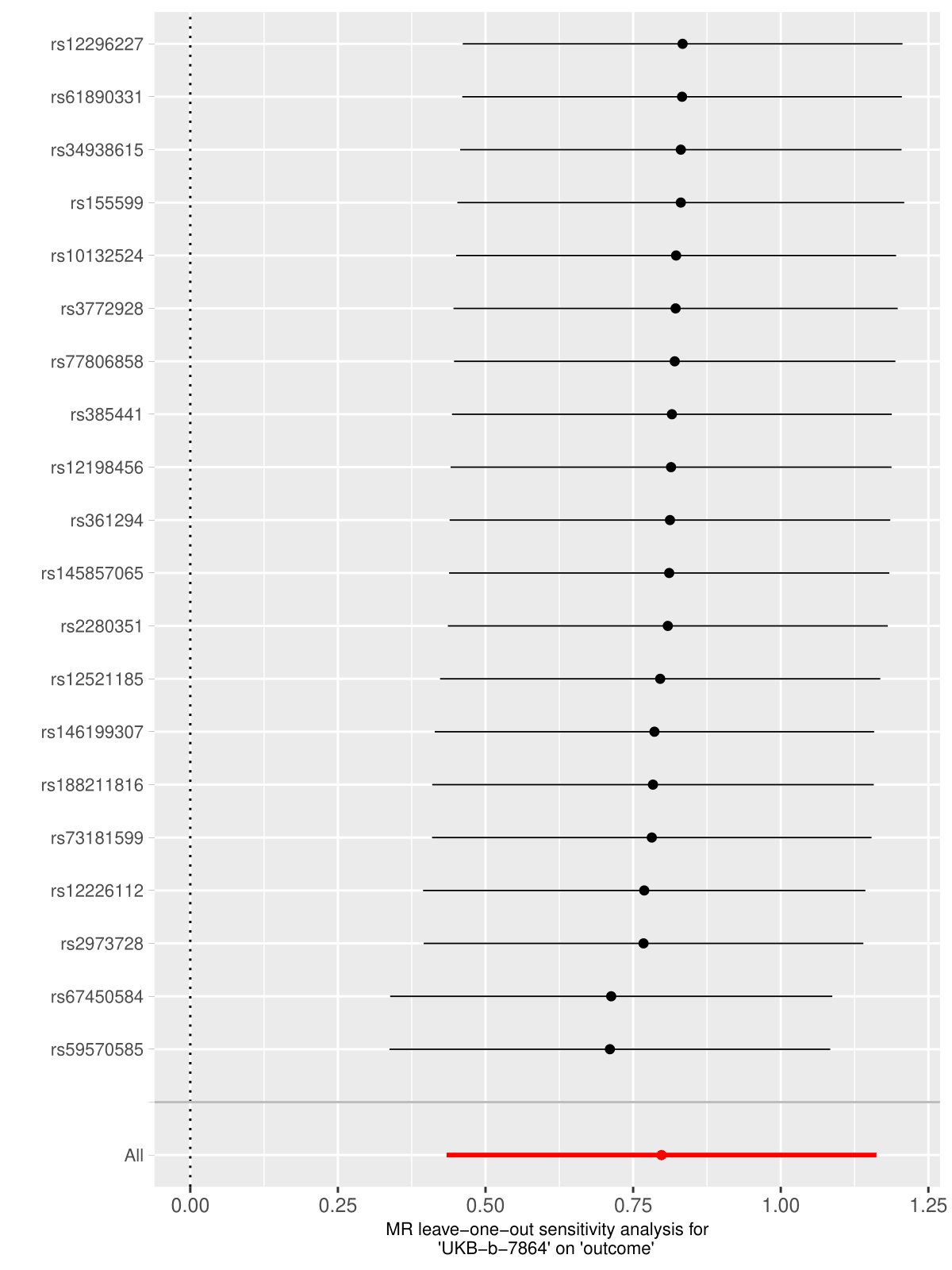
**

**Fig.S9**: leave-one-out plot of secondary post hoc analysis of vitamin B6 as risk factors on the risk of osteomyelitis (r2 < 0.001 within 10,000 kb windows and P ≤ 1E-05 and removing SNPs with a p<0.05 in the leave-one-out analysis)

**
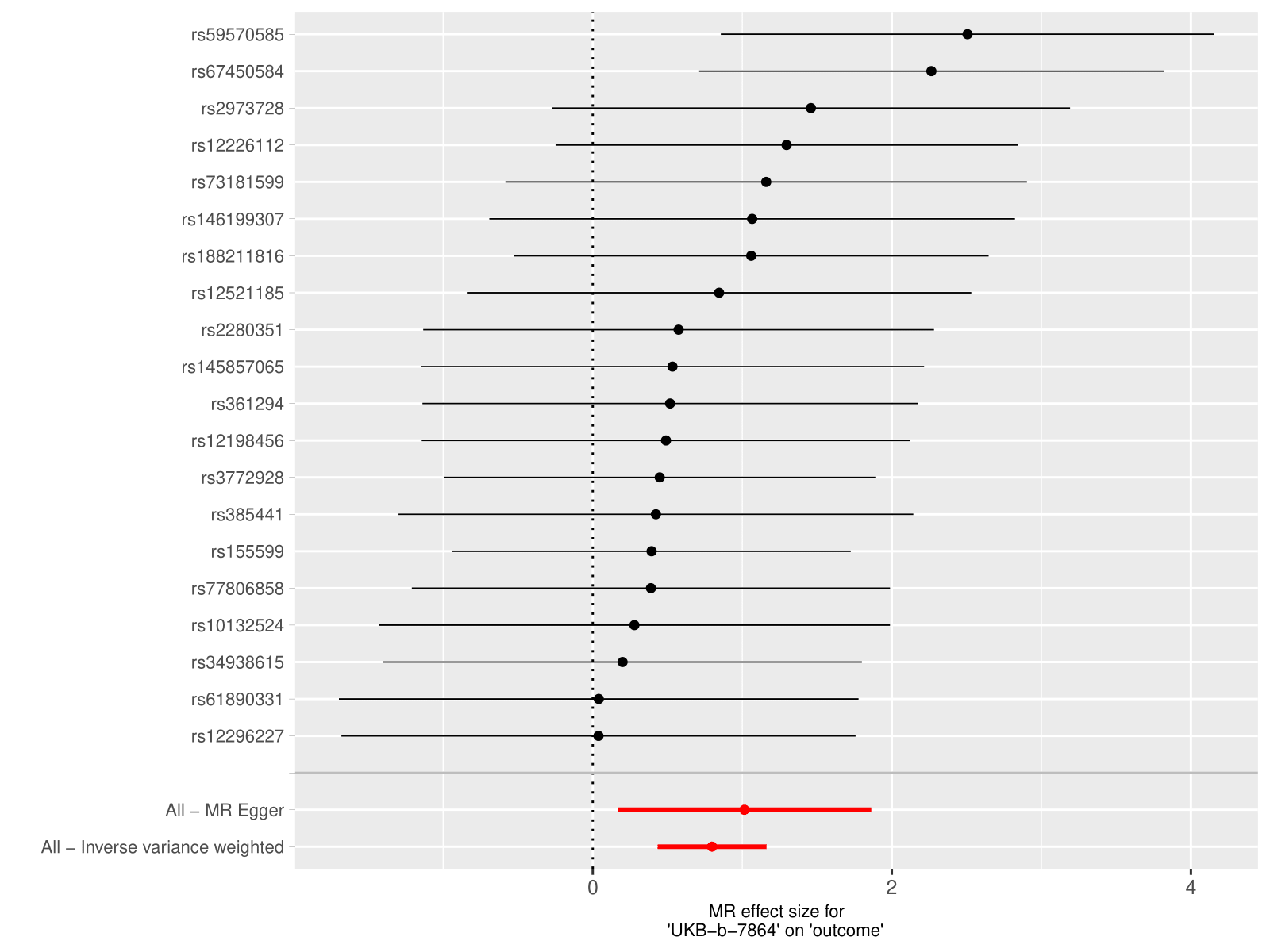
**

**Fig.S10**: Forest plot of secondary post hoc analysis of vitamin B6 as risk factors on the risk of osteomyelitis (r2 < 0.001 within 10,000 kb windows and P ≤ 1E-05 and removing SNPs with a p<0.05 in the leave-one-out analysis)

## Supplementary Tables

**Table S1.** ICD-10 codes for osteomyelitis infections

| Code | Description |
| --- | --- |
| M00 | Pyogenic arthritis |
| M00.0 | Staphylococcal arthritis and polyarthritis |
| M00.1 | Pneumococcal arthritis and polyarthritis |
| M00.2 | Other streptococcal arthritis and polyarthritis |
| M00.8 | Arthritis and polyarthritis due to other specified bacterial agents |
| M00.9 | Pyogenic arthritis, unspecified |
| M01 | Direct infections of joint in infectious and parasitic diseases classified elsewhere |
| M01.1 | Meningococcal arthritis |
| M01.1 | Tuberculous arthritis |
| M01.2 | Arthritis in Lyme disease |
| M01.3 | Arthritis in other bacterial diseases classified elsewhere |
| M01.4 | Rubella arthritis |
| M01.5 | Arthritis in other viral diseases classified elsewhere |
| M01.6 | Arthritis in mycoses |
| M01.8 | Arthritis in other infectious and parasitic diseases classified elsewhere |
| M46 | Other inflammatory spondylopathies |
| M46.0 | Spinal enthesopathy |
| M46.1 | Sacroiliitis, not elsewhere classified |
| M46.2 | Osteomyelitis of vertebra |
| M46.3 | Infection of intervertebral disc (pyogenic) |
| M46.4 | Discitis, unspecified |
| M46.5 | Other infective spondylopathies |
| M46.6 | Other specified inflammatory spondylopathies |
| M46.7 | Inflammatory spondylopathy, unspecified |
| M46.8 | Other osteomyelitis |
| M46.9 | Osteomyelitis, unspecified |
| M86.0 | Acute haematogenous osteomyelitis |
| M86.1 | Other acute osteomyelitis |
| M86.2 | Subacute osteomyelitis |
| M86.3 | Chronic multifocal osteomyelitis |
| M86.4 | Chronic osteomyelitis with draining sinus |
| M86.5 | Other chronic haematogenous osteomyelitis |
| M86.6 | Other chronic osteomyelitis |
| M86.8 | Other osteomyelitis |
| M86.9 | Osteomyelitis, unspecified |

**Table S2**. Genetic variants used as exposure for Mendelian randomization analyses.

| Exposure |  | SNP | EA | OA | Beta | Std | EAF | P | R2 | F |
| --- | --- | --- | --- | --- | --- | --- | --- | --- | --- | --- |
| Cu | Main analysis | rs1175550 | A | G | -0.198 | 0.032 | NA | 5.03E-10 | 0.0145 | 38 |
|  |  | rs2769264 | G | T | 0.313 | 0.034 | NA | 2.63E-20 | 0.0315 | 85 |
|  | Secondary analysis | rs1175550 | A | G | -0.198 | 0.032 | NA | 5.03E-10 | 0.0145 | 38 |
|  |  | rs2769264 | G | T | 0.313 | 0.034 | NA | 2.63E-20 | 0.0315 | 85 |
| SE QIMR | Main analysis | rs7700970 | C | T | -0.265 | 0.037 | NA | 7.17E-13 | 0.0193 | 51 |
|  | Secondary analysis | rs10023369 | A | G | -0.144 | 0.029 | NA | 4.42E-07 | 0.0094 | 25 |
|  |  | rs4950779 | C | T | 0.673 | 0.132 | NA | 3.16E-07 | 0.0099 | 26 |
|  |  | rs7700970 | C | T | -0.265 | 0.037 | NA | 7.17E-13 | 0.0193 | 51 |
| Se ALSPAC | Main analysis | rs921943 | C | T | 0.264 | 0.03 | NA | 1.43E-18 | 0.0289 | 77 |
|  | Secondary analysis | rs11948804 | C | T | 0.236 | 0.047 | NA | 3.90E-07 | 0.0096 | 25 |
|  |  | rs12951643 | A | G | 0.232 | 0.047 | NA | 8.01E-07 | 0.0093 | 24 |
|  |  | rs921943 | C | T | 0.264 | 0.03 | NA | 1.43E-18 | 0.0289 | 77 |
| Zn | Main analysis | rs1532423 | A | G | 0.178 | 0.026 | NA | 6.40E-12 | 0.0177 | 47 |
|  |  | rs2120019 | C | T | -0.287 | 0.033 | NA | 1.55E-18 | 0.0282 | 76 |
|  | Secondary analysis | rs11232535 | C | T | 0.325 | 0.065 | NA | 6.73E-07 | 0.0095 | 25 |
|  |  | rs11763353 | A | G | 0.192 | 0.039 | NA | 6.90E-07 | 0.0092 | 24 |
|  |  | rs1532423 | A | G | 0.178 | 0.026 | NA | 6.40E-12 | 0.0177 | 47 |
|  |  | rs2120019 | C | T | -0.287 | 0.033 | NA | 1.55E-18 | 0.0282 | 76 |
|  |  | rs7148590 | A | G | -0.14 | 0.026 | NA | 1.37E-07 | 0.0110 | 29 |
| Vitamin B6 | Main analysis | rs155599 | C | T | 0.034253 | 0.005985 | 0.705232 | 1.00E-08 | 0.0005 | 33 |
|  | Secondary analysis | rs12226112 | T | G | 0.028341 | 0.005727 | 0.341372 | 7.50E-07 | 0.0004 | 24 |
|  |  | rs155599 | C | T | 0.034253 | 0.005985 | 0.705232 | 1.00E-08 | 0.0005 | 33 |
|  |  | rs3772928 | C | T | -0.02924 | 0.005534 | 0.575413 | 1.30E-07 | 0.0004 | 28 |
|  |  | rs67450584 | T | C | 0.036705 | 0.007457 | 0.158007 | 8.60E-07 | 0.0004 | 24 |
| Vitamin B12 | Main analysis | rs1131603 | T | C | 0.132425 | 0.024152 | 0.046148 | 4.18E-08 | 0.0015 | 30 |
|  |  | rs12780845 | A | G | 0.066116 | 0.010749 | 0.316732 | 7.69E-10 | 0.0019 | 38 |
|  |  | rs1801222 | G | A | -0.09001 | 0.010416 | 0.355834 | 5.58E-18 | 0.0038 | 75 |
|  |  | rs4458686 | T | C | -0.06952 | 0.010392 | 0.373049 | 2.23E-11 | 0.0023 | 45 |
|  |  | rs506897 | C | G | -0.1084 | 0.010076 | 0.477601 | 5.43E-27 | 0.0059 | 116 |
|  |  | rs526934 | A | G | -0.084 | 0.011217 | 0.27799 | 6.92E-14 | 0.0029 | 56 |
|  | Secondary analysis | rs1131603 | T | C | 0.132425 | 0.024152 | 0.046148 | 4.18E-08 | 0.0015 | 30 |
|  |  | rs12780845 | A | G | 0.066116 | 0.010749 | 0.316732 | 7.69E-10 | 0.0019 | 38 |
|  |  | rs1801222 | G | A | -0.09001 | 0.010416 | 0.355834 | 5.58E-18 | 0.0038 | 75 |
|  |  | rs2972578 | A | G | -0.06251 | 0.012215 | 0.216098 | 3.09E-07 | 0.0014 | 26 |
|  |  | rs4458686 | T | C | -0.06952 | 0.010392 | 0.373049 | 2.23E-11 | 0.0023 | 45 |
|  |  | rs506897 | C | G | -0.1084 | 0.010076 | 0.477601 | 5.43E-27 | 0.0059 | 116 |
|  |  | rs526934 | A | G | -0.084 | 0.011217 | 0.27799 | 6.92E-14 | 0.0029 | 56 |
|  |  | rs61960878 | G | A | 0.086577 | 0.017624 | 0.089167 | 9.00E-07 | 0.0012 | 24 |
|  |  | rs79324875 | T | C | -0.12492 | 0.025045 | 0.042638 | 6.11E-07 | 0.0013 | 25 |
| Vitamin C | Main analysis | rs10051765 | T | C | -0.039 | 0.0066 | 0.6585 | 3.64E-09 | 0.0007 | 35 |
|  |  | rs10136000 | A | G | 0.0404 | 0.0071 | 0.2825 | 1.33E-08 | 0.0006 | 32 |
|  |  | rs117885456 | A | G | 0.0781 | 0.0116 | 0.0865 | 1.70E-11 | 0.0009 | 45 |
|  |  | rs12524110 | C | G | 0.0375 | 0.0063 | 0.5693 | 3.17E-09 | 0.0007 | 35 |
|  |  | rs13028225 | T | C | 0.1016 | 0.0089 | 0.8569 | 2.38E-30 | 0.0025 | 130 |
|  |  | rs174547 | T | C | -0.0364 | 0.0066 | 0.6721 | 3.84E-08 | 0.0006 | 30 |
|  |  | rs2559850 | A | G | 0.0583 | 0.0064 | 0.5979 | 6.30E-20 | 0.0016 | 83 |
|  |  | rs33972313 | T | C | -0.3601 | 0.0179 | 0.0319 | 4.61E-90 | 0.0077 | 405 |
|  |  | rs56738967 | C | G | 0.041 | 0.0067 | 0.321 | 7.62E-10 | 0.0007 | 37 |
|  |  | rs6693447 | T | G | 0.0393 | 0.0064 | 0.5509 | 6.25E-10 | 0.0007 | 38 |
|  |  | rs9895661 | T | C | 0.0625 | 0.0081 | 0.817 | 1.05E-14 | 0.0011 | 60 |
|  | Secondary analysis | rs10051765 | T | C | -0.039 | 0.0066 | 0.6585 | 3.64E-09 | 0.0007 | 35 |
|  |  | rs10136000 | A | G | 0.0404 | 0.0071 | 0.2825 | 1.33E-08 | 0.0006 | 32 |
|  |  | rs1165189 | A | C | -0.0378 | 0.0071 | 0.7469 | 1.18E-07 | 0.0005 | 28 |
|  |  | rs117885456 | A | G | 0.0781 | 0.0116 | 0.0865 | 1.70E-11 | 0.0009 | 45 |
|  |  | rs12524110 | C | G | 0.0375 | 0.0063 | 0.5693 | 3.17E-09 | 0.0007 | 35 |
|  |  | rs13028225 | T | C | 0.1016 | 0.0089 | 0.8569 | 2.38E-30 | 0.0025 | 130 |
|  |  | rs174547 | T | C | -0.0364 | 0.0066 | 0.6721 | 3.84E-08 | 0.0006 | 30 |
|  |  | rs2366388 | A | G | 0.0309 | 0.0063 | 0.4908 | 8.50E-07 | 0.0005 | 24 |
|  |  | rs2559850 | A | G | 0.0583 | 0.0064 | 0.5979 | 6.30E-20 | 0.0016 | 83 |
|  |  | rs2941484 | T | C | 0.0341 | 0.0063 | 0.4514 | 6.37E-08 | 0.0006 | 29 |
|  |  | rs33972313 | T | C | -0.3601 | 0.0179 | 0.0319 | 4.61E-90 | 0.0077 | 405 |
|  |  | rs56738967 | C | G | 0.041 | 0.0067 | 0.321 | 7.62E-10 | 0.0007 | 37 |
|  |  | rs6482188 | A | C | -0.0347 | 0.0068 | 0.3086 | 2.82E-07 | 0.0005 | 26 |
|  |  | rs6693447 | T | G | 0.0393 | 0.0064 | 0.5509 | 6.25E-10 | 0.0007 | 38 |
|  |  | rs676317 | T | C | -0.0366 | 0.0074 | 0.7264 | 7.33E-07 | 0.0005 | 24 |
|  |  | rs79234109 | A | G | 0.0453 | 0.0084 | 0.1617 | 7.70E-08 | 0.0006 | 29 |
|  |  | rs9895661 | T | C | 0.0625 | 0.0081 | 0.817 | 1.05E-14 | 0.0011 | 60 |
| Vitamin E | Main analysis |  |  |  |  |  |  |  |  |  |
|  | Seceondery analysis | rs111306778 | A | G | -0.04797 | 0.009572 | 0.089621 | 5.40E-07 | 0.0004 | 25 |
|  |  | rs12165526 | A | T | 0.047902 | 0.009178 | 0.100636 | 1.80E-07 | 0.0004 | 27 |
|  |  | rs4903544 | T | C | -0.02951 | 0.006021 | 0.30035 | 9.50E-07 | 0.0004 | 24 |
|  |  | rs536912 | A | C | 0.03046 | 0.006201 | 0.736049 | 9.00E-07 | 0.0004 | 24 |
|  |  | rs6033 | G | A | -0.05166 | 0.010556 | 0.072493 | 9.90E-07 | 0.0004 | 24 |
|  |  | rs71385328 | G | A | 0.130015 | 0.026206 | 0.011307 | 7.00E-07 | 0.0004 | 25 |
| Vitamin D | Main analysis | rs1011468 | A | G | -0.013795 | 0.001946 | 0.475748 | 1.42E-12 | 0.0001 | 50 |
|  |  | rs1047891 | A | C | -0.014168 | 0.002088 | 0.316449 | 1.19E-11 | 0.0001 | 46 |
|  |  | rs1048328 | A | G | 0.028256 | 0.003599 | 0.079719 | 4.36E-15 | 0.0001 | 62 |
|  |  | rs10771090 | G | A | -0.010681 | 0.00195 | 0.475398 | 4.38E-08 | 0.0001 | 30 |
|  |  | rs10859995 | C | T | -0.039396 | 0.001971 | 0.581322 | 7.56E-89 | 0.0008 | 400 |
|  |  | rs10864726 | T | C | 0.011792 | 0.001996 | 0.604499 | 3.59E-09 | 0.0001 | 35 |
|  |  | rs10887718 | T | C | -0.012476 | 0.001946 | 0.527362 | 1.50E-10 | 0.0001 | 41 |
|  |  | rs10908469 | C | A | 0.016044 | 0.002187 | 0.269894 | 2.30E-13 | 0.0001 | 54 |
|  |  | rs11127048 | A | G | 0.018107 | 0.002038 | 0.616569 | 6.69E-19 | 0.0002 | 79 |
|  |  | rs111529171 | C | G | -0.015488 | 0.002369 | 0.216376 | 6.41E-11 | 0.0001 | 43 |
|  |  | rs112285002 | T | C | 0.060321 | 0.002701 | 0.159529 | 2.09E-110 | 0.0010 | 499 |
|  |  | rs1137844 | G | C | -0.01457 | 0.002116 | 0.313364 | 5.90E-12 | 0.0001 | 47 |
|  |  | rs11500197 | A | G | -0.012229 | 0.002227 | 0.26183 | 4.09E-08 | 0.0001 | 30 |
|  |  | rs11542462 | A | G | -0.0218 | 0.002875 | 0.133941 | 3.56E-14 | 0.0001 | 57 |
|  |  | rs116472025 | A | G | 0.037298 | 0.006045 | 0.02789 | 7.02E-10 | 0.0001 | 38 |
|  |  | rs11846838 | A | G | -0.012996 | 0.002095 | 0.326092 | 5.74E-10 | 0.0001 | 38 |
|  |  | rs12123821 | T | C | 0.074472 | 0.004583 | 0.04793 | 2.52E-59 | 0.0005 | 264 |
|  |  | rs12196316 | C | T | -0.01242 | 0.00213 | 0.301136 | 5.68E-09 | 0.0001 | 34 |
|  |  | rs12278461 | T | C | 0.105426 | 0.002355 | 0.781517 | 1.00E-200 | 0.0042 | 2004 |
|  |  | rs1229984 | C | T | -0.046854 | 0.006481 | 0.973099 | 5.07E-13 | 0.0001 | 52 |
|  |  | rs12317268 | G | A | -0.018528 | 0.002717 | 0.151589 | 9.47E-12 | 0.0001 | 47 |
|  |  | rs12462826 | A | G | -0.011799 | 0.002029 | 0.371101 | 6.28E-09 | 0.0001 | 34 |
|  |  | rs13022972 | G | A | -0.012662 | 0.002185 | 0.545302 | 6.98E-09 | 0.0001 | 34 |
|  |  | rs13104531 | T | C | -0.01872 | 0.002634 | 0.16309 | 1.23E-12 | 0.0001 | 51 |
|  |  | rs13245123 | A | C | 0.01293 | 0.002218 | 0.638444 | 5.71E-09 | 0.0001 | 34 |
|  |  | rs13284054 | C | T | 0.01727 | 0.002998 | 0.124389 | 8.60E-09 | 0.0001 | 33 |
|  |  | rs1660818 | A | G | 0.013553 | 0.002069 | 0.323225 | 5.94E-11 | 0.0001 | 43 |
|  |  | rs1714363 | T | C | 0.012391 | 0.002206 | 0.261499 | 1.99E-08 | 0.0001 | 32 |
|  |  | rs174418 | C | T | 0.021901 | 0.001987 | 0.595318 | 3.20E-28 | 0.0003 | 121 |
|  |  | rs17651741 | A | G | -0.013446 | 0.002465 | 0.193832 | 4.98E-08 | 0.0001 | 30 |
|  |  | rs1800588 | T | C | -0.029769 | 0.002366 | 0.21461 | 2.98E-36 | 0.0003 | 158 |
|  |  | rs1800775 | A | C | -0.016619 | 0.00195 | 0.486317 | 1.64E-17 | 0.0002 | 73 |
|  |  | rs187245277 | A | G | -0.072217 | 0.009008 | 0.012806 | 1.14E-15 | 0.0001 | 64 |
|  |  | rs1950039 | C | T | -0.014612 | 0.001983 | 0.60732 | 1.78E-13 | 0.0001 | 54 |
|  |  | rs1972994 | T | A | -0.017509 | 0.002036 | 0.64702 | 8.52E-18 | 0.0002 | 74 |
|  |  | rs2012736 | A | C | -0.045584 | 0.003595 | 0.08152 | 8.46E-37 | 0.0003 | 161 |
|  |  | rs2037511 | A | G | 0.016025 | 0.002618 | 0.165391 | 9.60E-10 | 0.0001 | 37 |
|  |  | rs2074735 | C | G | 0.027256 | 0.003969 | 0.064197 | 6.82E-12 | 0.0001 | 47 |
|  |  | rs2144530 | T | C | -0.031755 | 0.002546 | 0.82006 | 1.15E-35 | 0.0003 | 156 |
|  |  | rs2229742 | C | G | -0.025738 | 0.00319 | 0.103928 | 7.50E-16 | 0.0001 | 65 |
|  |  | rs2245133 | C | T | -0.020883 | 0.002621 | 0.16631 | 1.69E-15 | 0.0001 | 63 |
|  |  | rs257380 | A | T | 0.011786 | 0.001946 | 0.519531 | 1.43E-09 | 0.0001 | 37 |
|  |  | rs2762938 | A | G | 0.01327 | 0.002016 | 0.585371 | 4.84E-11 | 0.0001 | 43 |
|  |  | rs27774 | A | G | -0.012072 | 0.002138 | 0.292475 | 1.68E-08 | 0.0001 | 32 |
|  |  | rs28393895 | C | T | 0.073208 | 0.002061 | 0.453615 | 1.00E-200 | 0.0026 | 1262 |
|  |  | rs2847500 | A | G | -0.021129 | 0.002949 | 0.124391 | 8.05E-13 | 0.0001 | 51 |
|  |  | rs2909218 | T | C | 0.016894 | 0.002418 | 0.792744 | 2.91E-12 | 0.0001 | 49 |
|  |  | rs2934744 | C | A | 0.022405 | 0.002119 | 0.356454 | 4.37E-26 | 0.0003 | 112 |
|  |  | rs34726834 | T | C | 0.013824 | 0.002239 | 0.253615 | 6.90E-10 | 0.0001 | 38 |
|  |  | rs36037728 | T | C | -0.05506 | 0.006074 | 0.026323 | 1.32E-19 | 0.0002 | 82 |
|  |  | rs373397810 | T | C | -0.016002 | 0.002703 | 0.815011 | 3.29E-09 | 0.0001 | 35 |
|  |  | rs3750296 | C | G | -0.020818 | 0.002042 | 0.341423 | 2.29E-24 | 0.0002 | 104 |
|  |  | rs4121823 | A | T | -0.016268 | 0.002681 | 0.838088 | 1.34E-09 | 0.0001 | 37 |
|  |  | rs4267257 | G | A | -0.014447 | 0.002595 | 0.185272 | 2.65E-08 | 0.0001 | 31 |
|  |  | rs4364259 | A | G | 0.015798 | 0.002422 | 0.205042 | 7.14E-11 | 0.0001 | 43 |
|  |  | rs4616820 | T | C | -0.01104 | 0.001946 | 0.465968 | 1.43E-08 | 0.0001 | 32 |
|  |  | rs4635554 | G | T | -0.012711 | 0.002057 | 0.336358 | 6.63E-10 | 0.0001 | 38 |
|  |  | rs466360 | A | G | -0.011398 | 0.001996 | 0.392078 | 1.16E-08 | 0.0001 | 33 |
|  |  | rs5112 | G | C | -0.014854 | 0.002114 | 0.532421 | 2.18E-12 | 0.0001 | 49 |
|  |  | rs523583 | C | A | 0.012174 | 0.001963 | 0.469219 | 5.76E-10 | 0.0001 | 38 |
|  |  | rs532436 | A | G | -0.015051 | 0.002515 | 0.184388 | 2.24E-09 | 0.0001 | 36 |
|  |  | rs55886116 | T | G | 0.014479 | 0.002478 | 0.200874 | 5.27E-09 | 0.0001 | 34 |
|  |  | rs58542926 | T | C | 0.032488 | 0.00367 | 0.075847 | 9.14E-19 | 0.0002 | 78 |
|  |  | rs6127099 | T | A | -0.036797 | 0.002219 | 0.279034 | 9.91E-62 | 0.0006 | 275 |
|  |  | rs61747728 | T | C | 0.031656 | 0.005074 | 0.038151 | 4.54E-10 | 0.0001 | 39 |
|  |  | rs61815559 | T | A | 0.083994 | 0.00553 | 0.033867 | 4.70E-52 | 0.0005 | 231 |
|  |  | rs6438900 | G | C | 0.013584 | 0.002221 | 0.260706 | 9.87E-10 | 0.0001 | 37 |
|  |  | rs6698680 | G | A | -0.011928 | 0.001947 | 0.464195 | 9.21E-10 | 0.0001 | 38 |
|  |  | rs6724965 | G | A | -0.016541 | 0.002573 | 0.171608 | 1.33E-10 | 0.0001 | 41 |
|  |  | rs6773343 | T | C | 0.01268 | 0.002171 | 0.720232 | 5.37E-09 | 0.0001 | 34 |
|  |  | rs6954700 | A | G | -0.011159 | 0.002006 | 0.392005 | 2.71E-08 | 0.0001 | 31 |
|  |  | rs71383766 | T | C | 0.012569 | 0.002065 | 0.419574 | 1.18E-09 | 0.0001 | 37 |
|  |  | rs7178572 | G | A | -0.01449 | 0.002151 | 0.711051 | 1.70E-11 | 0.0001 | 45 |
|  |  | rs72632970 | T | G | -0.089747 | 0.003679 | 0.075108 | 2.30E-131 | 0.0012 | 595 |
|  |  | rs72645665 | A | G | -0.086778 | 0.002456 | 0.19152 | 1.00E-200 | 0.0026 | 1248 |
|  |  | rs72665698 | G | T | 0.013976 | 0.00233 | 0.223334 | 2.06E-09 | 0.0001 | 36 |
|  |  | rs72897736 | A | T | 0.017369 | 0.002953 | 0.129967 | 4.18E-09 | 0.0001 | 35 |
|  |  | rs73015021 | G | A | 0.023034 | 0.002983 | 0.121031 | 1.21E-14 | 0.0001 | 60 |
|  |  | rs7314285 | G | T | 0.021685 | 0.003894 | 0.068219 | 2.62E-08 | 0.0001 | 31 |
|  |  | rs7439098 | A | G | -0.026848 | 0.001932 | 0.471869 | 7.07E-44 | 0.0004 | 193 |
|  |  | rs7519574 | A | G | 0.016991 | 0.002536 | 0.181694 | 2.17E-11 | 0.0001 | 45 |
|  |  | rs7528419 | G | A | 0.019031 | 0.002321 | 0.224958 | 2.56E-16 | 0.0001 | 67 |
|  |  | rs7569755 | A | G | 0.013923 | 0.002142 | 0.292374 | 8.34E-11 | 0.0001 | 42 |
|  |  | rs7650253 | A | T | 0.014562 | 0.002282 | 0.689684 | 1.82E-10 | 0.0001 | 41 |
|  |  | rs7691791 | T | C | -0.014182 | 0.002112 | 0.297894 | 1.94E-11 | 0.0001 | 45 |
|  |  | rs7718395 | G | C | 0.012632 | 0.002096 | 0.319522 | 1.72E-09 | 0.0001 | 36 |
|  |  | rs7724488 | G | A | 0.011689 | 0.001988 | 0.432626 | 4.25E-09 | 0.0001 | 35 |
|  |  | rs77532868 | T | C | 0.025802 | 0.00431 | 0.053725 | 2.21E-09 | 0.0001 | 36 |
|  |  | rs77924615 | A | G | -0.015792 | 0.002464 | 0.197773 | 1.52E-10 | 0.0001 | 41 |
|  |  | rs7828742 | G | A | -0.02193 | 0.00199 | 0.596853 | 3.40E-28 | 0.0003 | 121 |
|  |  | rs78644250 | G | C | -0.014573 | 0.002651 | 0.18209 | 3.94E-08 | 0.0001 | 30 |
|  |  | rs78649910 | A | T | -0.018331 | 0.003122 | 0.110004 | 4.41E-09 | 0.0001 | 34 |
|  |  | rs7938266 | G | A | -0.067817 | 0.001972 | 0.410108 | 1.00E-200 | 0.0025 | 1183 |
|  |  | rs804280 | A | C | 0.013033 | 0.001978 | 0.581959 | 4.61E-11 | 0.0001 | 43 |
|  |  | rs8063706 | T | A | 0.012968 | 0.002198 | 0.272828 | 3.72E-09 | 0.0001 | 35 |
|  |  | rs8091117 | A | C | -0.024071 | 0.003943 | 0.065396 | 1.06E-09 | 0.0001 | 37 |
|  |  | rs814573 | T | A | -0.01508 | 0.002642 | 0.183339 | 1.18E-08 | 0.0001 | 33 |
|  |  | rs867772 | G | A | -0.013838 | 0.002091 | 0.681808 | 3.75E-11 | 0.0001 | 44 |
|  |  | rs9423639 | T | C | -0.011997 | 0.002185 | 0.274575 | 4.08E-08 | 0.0001 | 30 |
|  |  | rs942380 | G | A | 0.011434 | 0.001981 | 0.594833 | 8.10E-09 | 0.0001 | 33 |
|  |  | rs9476310 | T | C | 0.011133 | 0.001948 | 0.511302 | 1.13E-08 | 0.0001 | 33 |
|  |  | rs9536961 | G | A | -0.011759 | 0.00207 | 0.346767 | 1.37E-08 | 0.0001 | 32 |
|  |  | rs960596 | T | C | 0.012414 | 0.002076 | 0.339588 | 2.29E-09 | 0.0001 | 36 |
|  |  | rs964184 | C | G | 0.03977 | 0.002858 | 0.863547 | 5.73E-44 | 0.0004 | 194 |
|  |  | rs9668081 | T | C | 0.0116 | 0.001988 | 0.47058 | 5.49E-09 | 0.0001 | 34 |
|  | Secondary analysis | rs10022559 | T | A | 0.02126 | 0.002022 | 0.518069 | 7.79E-26 | 0.0002 | 111 |
|  |  | rs10101205 | C | T | 0.013764 | 0.002679 | 0.839956 | 2.83E-07 | 0.0001 | 26 |
|  |  | rs1011468 | A | G | -0.013795 | 0.001946 | 0.475748 | 1.42E-12 | 0.0001 | 50 |
|  |  | rs10128681 | C | T | -0.068486 | 0.001965 | 0.410204 | 1.00E-200 | 0.0025 | 1215 |
|  |  | rs1047891 | A | C | -0.014168 | 0.002088 | 0.316449 | 1.19E-11 | 0.0001 | 46 |
|  |  | rs1048328 | A | G | 0.028256 | 0.003599 | 0.079719 | 4.36E-15 | 0.0001 | 62 |
|  |  | rs10758321 | A | G | 0.010066 | 0.00198 | 0.414546 | 3.79E-07 | 0.0001 | 26 |
|  |  | rs10771090 | G | A | -0.010681 | 0.00195 | 0.475398 | 4.38E-08 | 0.0001 | 30 |
|  |  | rs10794655 | T | A | 0.011032 | 0.002241 | 0.25147 | 8.65E-07 | 0.0001 | 24 |
|  |  | rs10859995 | C | T | -0.039396 | 0.001971 | 0.581322 | 7.56E-89 | 0.0008 | 400 |
|  |  | rs10864726 | T | C | 0.011792 | 0.001996 | 0.604499 | 3.59E-09 | 0.0001 | 35 |
|  |  | rs10887718 | T | C | -0.012476 | 0.001946 | 0.527362 | 1.50E-10 | 0.0001 | 41 |
|  |  | rs10896045 | G | A | 0.010475 | 0.002121 | 0.701095 | 8.06E-07 | 0.0001 | 24 |
|  |  | rs10898241 | C | T | -0.100267 | 0.002877 | 0.131936 | 1.00E-200 | 0.0025 | 1215 |
|  |  | rs10908469 | C | A | 0.016044 | 0.002187 | 0.269894 | 2.30E-13 | 0.0001 | 54 |
|  |  | rs11057274 | A | C | -0.032076 | 0.006225 | 0.026185 | 2.62E-07 | 0.0001 | 27 |
|  |  | rs11127048 | A | G | 0.018107 | 0.002038 | 0.616569 | 6.69E-19 | 0.0002 | 79 |
|  |  | rs111529171 | C | G | -0.015488 | 0.002369 | 0.216376 | 6.41E-11 | 0.0001 | 43 |
|  |  | rs11209952 | T | C | -0.010515 | 0.001973 | 0.592862 | 1.01E-07 | 0.0001 | 28 |
|  |  | rs112285002 | T | C | 0.060321 | 0.002701 | 0.159529 | 2.09E-110 | 0.0010 | 499 |
|  |  | rs112736716 | C | T | 0.010624 | 0.002085 | 0.600117 | 3.55E-07 | 0.0001 | 26 |
|  |  | rs112989220 | A | G | 0.010025 | 0.001989 | 0.450164 | 4.76E-07 | 0.0001 | 25 |
|  |  | rs113209890 | T | C | -0.050478 | 0.003219 | 0.102811 | 2.30E-55 | 0.0005 | 246 |
|  |  | rs1137844 | G | C | -0.01457 | 0.002116 | 0.313364 | 5.90E-12 | 0.0001 | 47 |
|  |  | rs11500197 | A | G | -0.012229 | 0.002227 | 0.26183 | 4.09E-08 | 0.0001 | 30 |
|  |  | rs11542462 | A | G | -0.0218 | 0.002875 | 0.133941 | 3.56E-14 | 0.0001 | 57 |
|  |  | rs11602347 | G | C | 0.01011 | 0.001996 | 0.407148 | 4.18E-07 | 0.0001 | 26 |
|  |  | rs11624558 | C | T | -0.010546 | 0.002133 | 0.303654 | 7.78E-07 | 0.0001 | 24 |
|  |  | rs116472025 | A | G | 0.037298 | 0.006045 | 0.02789 | 7.02E-10 | 0.0001 | 38 |
|  |  | rs117862422 | C | T | -0.042589 | 0.00811 | 0.015717 | 1.54E-07 | 0.0001 | 28 |
|  |  | rs117962481 | A | T | 0.011155 | 0.002124 | 0.350417 | 1.54E-07 | 0.0001 | 28 |
|  |  | rs11846838 | A | G | -0.012996 | 0.002095 | 0.326092 | 5.74E-10 | 0.0001 | 38 |
|  |  | rs11885466 | T | C | -0.018832 | 0.003698 | 0.075285 | 3.61E-07 | 0.0001 | 26 |
|  |  | rs12123821 | T | C | 0.074472 | 0.004583 | 0.04793 | 2.52E-59 | 0.0005 | 264 |
|  |  | rs1214761 | G | A | 0.010314 | 0.002079 | 0.676756 | 7.16E-07 | 0.0001 | 25 |
|  |  | rs12196316 | C | T | -0.01242 | 0.00213 | 0.301136 | 5.68E-09 | 0.0001 | 34 |
|  |  | rs1229984 | C | T | -0.046854 | 0.006481 | 0.973099 | 5.07E-13 | 0.0001 | 52 |
|  |  | rs12317268 | G | A | -0.018528 | 0.002717 | 0.151589 | 9.47E-12 | 0.0001 | 47 |
|  |  | rs12462826 | A | G | -0.011799 | 0.002029 | 0.371101 | 6.28E-09 | 0.0001 | 34 |
|  |  | rs12554549 | T | C | 0.020931 | 0.004003 | 0.065186 | 1.75E-07 | 0.0001 | 27 |
|  |  | rs12964203 | C | T | -0.010946 | 0.002215 | 0.265139 | 7.88E-07 | 0.0001 | 24 |
|  |  | rs13022972 | G | A | -0.012662 | 0.002185 | 0.545302 | 6.98E-09 | 0.0001 | 34 |
|  |  | rs13030535 | T | C | 0.01021 | 0.001988 | 0.392554 | 2.89E-07 | 0.0001 | 26 |
|  |  | rs13123929 | A | G | 0.01154 | 0.002307 | 0.767187 | 5.75E-07 | 0.0001 | 25 |
|  |  | rs13137545 | A | G | -0.014 | 0.002022 | 0.624613 | 4.61E-12 | 0.0001 | 48 |
|  |  | rs13245123 | A | C | 0.01293 | 0.002218 | 0.638444 | 5.71E-09 | 0.0001 | 34 |
|  |  | rs13278404 | G | C | 0.014618 | 0.002834 | 0.857704 | 2.55E-07 | 0.0001 | 27 |
|  |  | rs13284054 | C | T | 0.01727 | 0.002998 | 0.124389 | 8.60E-09 | 0.0001 | 33 |
|  |  | rs1484423 | G | A | 0.011503 | 0.00221 | 0.25993 | 1.98E-07 | 0.0001 | 27 |
|  |  | rs1542926 | C | T | 0.016355 | 0.003067 | 0.117024 | 9.91E-08 | 0.0001 | 28 |
|  |  | rs1714363 | T | C | 0.012391 | 0.002206 | 0.261499 | 1.99E-08 | 0.0001 | 32 |
|  |  | rs17161467 | A | C | -0.050751 | 0.00509 | 0.037885 | 2.19E-23 | 0.0002 | 99 |
|  |  | rs174418 | C | T | 0.021901 | 0.001987 | 0.595318 | 3.20E-28 | 0.0003 | 121 |
|  |  | rs17616063 | G | A | 0.0182 | 0.00371 | 0.075428 | 9.51E-07 | 0.0000 | 24 |
|  |  | rs17651741 | A | G | -0.013446 | 0.002465 | 0.193832 | 4.98E-08 | 0.0001 | 30 |
|  |  | rs1790337 | C | T | 0.092443 | 0.005028 | 0.958232 | 1.99E-75 | 0.0007 | 338 |
|  |  | rs1800588 | T | C | -0.029769 | 0.002366 | 0.21461 | 2.98E-36 | 0.0003 | 158 |
|  |  | rs1800775 | A | C | -0.016619 | 0.00195 | 0.486317 | 1.64E-17 | 0.0002 | 73 |
|  |  | rs183717655 | C | G | -0.013725 | 0.002676 | 0.180459 | 2.98E-07 | 0.0001 | 26 |
|  |  | rs1843096 | A | G | 0.012206 | 0.002432 | 0.224461 | 5.29E-07 | 0.0001 | 25 |
|  |  | rs1883711 | C | G | -0.030854 | 0.005755 | 0.031203 | 8.45E-08 | 0.0001 | 29 |
|  |  | rs1909585 | T | C | 0.0105 | 0.002071 | 0.345901 | 4.08E-07 | 0.0001 | 26 |
|  |  | rs1950039 | C | T | -0.014612 | 0.001983 | 0.60732 | 1.78E-13 | 0.0001 | 54 |
|  |  | rs1972994 | T | A | -0.017509 | 0.002036 | 0.64702 | 8.52E-18 | 0.0002 | 74 |
|  |  | rs2012736 | A | C | -0.045584 | 0.003595 | 0.08152 | 8.46E-37 | 0.0003 | 161 |
|  |  | rs2037511 | A | G | 0.016025 | 0.002618 | 0.165391 | 9.60E-10 | 0.0001 | 37 |
|  |  | rs2074735 | C | G | 0.027256 | 0.003969 | 0.064197 | 6.82E-12 | 0.0001 | 47 |
|  |  | rs2144530 | T | C | -0.031755 | 0.002546 | 0.82006 | 1.15E-35 | 0.0003 | 156 |
|  |  | rs2157829 | G | A | -0.010282 | 0.002063 | 0.664366 | 6.33E-07 | 0.0001 | 25 |
|  |  | rs2229742 | C | G | -0.025738 | 0.00319 | 0.103928 | 7.50E-16 | 0.0001 | 65 |
|  |  | rs2245133 | C | T | -0.020883 | 0.002621 | 0.16631 | 1.69E-15 | 0.0001 | 63 |
|  |  | rs2334097 | G | A | -0.01095 | 0.002151 | 0.305283 | 3.64E-07 | 0.0001 | 26 |
|  |  | rs2544725 | C | A | -0.009785 | 0.001976 | 0.590063 | 7.51E-07 | 0.0001 | 25 |
|  |  | rs257380 | A | T | 0.011786 | 0.001946 | 0.519531 | 1.43E-09 | 0.0001 | 37 |
|  |  | rs2647463 | C | T | -0.010842 | 0.002049 | 0.347461 | 1.24E-07 | 0.0001 | 28 |
|  |  | rs2762938 | A | G | 0.01327 | 0.002016 | 0.585371 | 4.84E-11 | 0.0001 | 43 |
|  |  | rs27774 | A | G | -0.012072 | 0.002138 | 0.292475 | 1.68E-08 | 0.0001 | 32 |
|  |  | rs28437159 | T | C | 0.056646 | 0.002683 | 0.154236 | 6.89E-99 | 0.0009 | 446 |
|  |  | rs2847500 | A | G | -0.021129 | 0.002949 | 0.124391 | 8.05E-13 | 0.0001 | 51 |
|  |  | rs28798705 | G | A | -0.012263 | 0.002264 | 0.315057 | 6.21E-08 | 0.0001 | 29 |
|  |  | rs2909218 | T | C | 0.016894 | 0.002418 | 0.792744 | 2.91E-12 | 0.0001 | 49 |
|  |  | rs2934744 | C | A | 0.022405 | 0.002119 | 0.356454 | 4.37E-26 | 0.0003 | 112 |
|  |  | rs34560261 | T | C | 0.013552 | 0.002657 | 0.170233 | 3.45E-07 | 0.0001 | 26 |
|  |  | rs34726834 | T | C | 0.013824 | 0.002239 | 0.253615 | 6.90E-10 | 0.0001 | 38 |
|  |  | rs35535415 | C | G | -0.013927 | 0.00281 | 0.141388 | 7.33E-07 | 0.0001 | 25 |
|  |  | rs35846253 | T | C | -0.060836 | 0.002478 | 0.189054 | 4.66E-133 | 0.0012 | 603 |
|  |  | rs373397810 | T | C | -0.016002 | 0.002703 | 0.815011 | 3.29E-09 | 0.0001 | 35 |
|  |  | rs3750296 | C | G | -0.020818 | 0.002042 | 0.341423 | 2.29E-24 | 0.0002 | 104 |
|  |  | rs4121823 | A | T | -0.016268 | 0.002681 | 0.838088 | 1.34E-09 | 0.0001 | 37 |
|  |  | rs4267257 | G | A | -0.014447 | 0.002595 | 0.185272 | 2.65E-08 | 0.0001 | 31 |
|  |  | rs4364259 | A | G | 0.015798 | 0.002422 | 0.205042 | 7.14E-11 | 0.0001 | 43 |
|  |  | rs4553272 | T | C | -0.01036 | 0.001958 | 0.477296 | 1.24E-07 | 0.0001 | 28 |
|  |  | rs4603973 | G | T | -0.012304 | 0.00226 | 0.680688 | 5.30E-08 | 0.0001 | 30 |
|  |  | rs4616820 | T | C | -0.01104 | 0.001946 | 0.465968 | 1.43E-08 | 0.0001 | 32 |
|  |  | rs4635554 | G | T | -0.012711 | 0.002057 | 0.336358 | 6.63E-10 | 0.0001 | 38 |
|  |  | rs466360 | A | G | -0.011398 | 0.001996 | 0.392078 | 1.16E-08 | 0.0001 | 33 |
|  |  | rs4738679 | A | G | 0.010677 | 0.002062 | 0.665078 | 2.28E-07 | 0.0001 | 27 |
|  |  | rs4788475 | G | T | 0.022223 | 0.004341 | 0.943611 | 3.13E-07 | 0.0001 | 26 |
|  |  | rs499974 | A | C | 0.016832 | 0.002648 | 0.158029 | 2.12E-10 | 0.0001 | 40 |
|  |  | rs5112 | G | C | -0.014854 | 0.002114 | 0.532421 | 2.18E-12 | 0.0001 | 49 |
|  |  | rs512083 | C | T | 0.009979 | 0.001948 | 0.460665 | 3.07E-07 | 0.0001 | 26 |
|  |  | rs532436 | A | G | -0.015051 | 0.002515 | 0.184388 | 2.24E-09 | 0.0001 | 36 |
|  |  | rs55886116 | T | G | 0.014479 | 0.002478 | 0.200874 | 5.27E-09 | 0.0001 | 34 |
|  |  | rs56185965 | A | T | 0.013665 | 0.002727 | 0.161333 | 5.51E-07 | 0.0001 | 25 |
|  |  | rs56257628 | A | C | -0.016045 | 0.003036 | 0.11864 | 1.28E-07 | 0.0001 | 28 |
|  |  | rs58542926 | T | C | 0.032488 | 0.00367 | 0.075847 | 9.14E-19 | 0.0002 | 78 |
|  |  | rs59789656 | T | C | -0.012473 | 0.002297 | 0.246224 | 5.80E-08 | 0.0001 | 29 |
|  |  | rs6127099 | T | A | -0.036797 | 0.002219 | 0.279034 | 9.91E-62 | 0.0006 | 275 |
|  |  | rs61747728 | T | C | 0.031656 | 0.005074 | 0.038151 | 4.54E-10 | 0.0001 | 39 |
|  |  | rs61815559 | T | A | 0.083994 | 0.00553 | 0.033867 | 4.70E-52 | 0.0005 | 231 |
|  |  | rs61887421 | C | T | -0.032911 | 0.005491 | 0.034104 | 2.12E-09 | 0.0001 | 36 |
|  |  | rs6438900 | G | C | 0.013584 | 0.002221 | 0.260706 | 9.87E-10 | 0.0001 | 37 |
|  |  | rs6698680 | G | A | -0.011928 | 0.001947 | 0.464195 | 9.21E-10 | 0.0001 | 38 |
|  |  | rs6724965 | G | A | -0.016541 | 0.002573 | 0.171608 | 1.33E-10 | 0.0001 | 41 |
|  |  | rs6773343 | T | C | 0.01268 | 0.002171 | 0.720232 | 5.37E-09 | 0.0001 | 34 |
|  |  | rs6858160 | G | A | 0.074722 | 0.00203 | 0.652733 | 1.00E-200 | 0.0028 | 1355 |
|  |  | rs6954700 | A | G | -0.011159 | 0.002006 | 0.392005 | 2.71E-08 | 0.0001 | 31 |
|  |  | rs71383766 | T | C | 0.012569 | 0.002065 | 0.419574 | 1.18E-09 | 0.0001 | 37 |
|  |  | rs7178572 | G | A | -0.01449 | 0.002151 | 0.711051 | 1.70E-11 | 0.0001 | 45 |
|  |  | rs7204844 | A | G | 0.009777 | 0.001987 | 0.395777 | 8.75E-07 | 0.0001 | 24 |
|  |  | rs72631431 | T | C | 0.01069 | 0.002124 | 0.313247 | 4.91E-07 | 0.0001 | 25 |
|  |  | rs72632970 | T | G | -0.089747 | 0.003679 | 0.075108 | 2.30E-131 | 0.0012 | 595 |
|  |  | rs72665698 | G | T | 0.013976 | 0.00233 | 0.223334 | 2.06E-09 | 0.0001 | 36 |
|  |  | rs72680101 | A | G | -0.027569 | 0.005123 | 0.038707 | 7.54E-08 | 0.0001 | 29 |
|  |  | rs72897736 | A | T | 0.017369 | 0.002953 | 0.129967 | 4.18E-09 | 0.0001 | 35 |
|  |  | rs73015021 | G | A | 0.023034 | 0.002983 | 0.121031 | 1.21E-14 | 0.0001 | 60 |
|  |  | rs73030358 | G | A | -0.013085 | 0.002635 | 0.173716 | 6.95E-07 | 0.0001 | 25 |
|  |  | rs7314285 | G | T | 0.021685 | 0.003894 | 0.068219 | 2.62E-08 | 0.0001 | 31 |
|  |  | rs745797 | A | G | -0.014886 | 0.002924 | 0.129404 | 3.61E-07 | 0.0001 | 26 |
|  |  | rs74797364 | T | G | 0.020229 | 0.002851 | 0.159298 | 1.35E-12 | 0.0001 | 50 |
|  |  | rs7519574 | A | G | 0.016991 | 0.002536 | 0.181694 | 2.17E-11 | 0.0001 | 45 |
|  |  | rs7528419 | G | A | 0.019031 | 0.002321 | 0.224958 | 2.56E-16 | 0.0001 | 67 |
|  |  | rs7569755 | A | G | 0.013923 | 0.002142 | 0.292374 | 8.34E-11 | 0.0001 | 42 |
|  |  | rs75936148 | A | G | -0.017158 | 0.003328 | 0.098643 | 2.58E-07 | 0.0001 | 27 |
|  |  | rs7650253 | A | T | 0.014562 | 0.002282 | 0.689684 | 1.82E-10 | 0.0001 | 41 |
|  |  | rs7691791 | T | C | -0.014182 | 0.002112 | 0.297894 | 1.94E-11 | 0.0001 | 45 |
|  |  | rs7718395 | G | C | 0.012632 | 0.002096 | 0.319522 | 1.72E-09 | 0.0001 | 36 |
|  |  | rs77194050 | G | A | 0.023163 | 0.004447 | 0.052229 | 1.94E-07 | 0.0001 | 27 |
|  |  | rs7724488 | G | A | 0.011689 | 0.001988 | 0.432626 | 4.25E-09 | 0.0001 | 35 |
|  |  | rs77532868 | T | C | 0.025802 | 0.00431 | 0.053725 | 2.21E-09 | 0.0001 | 36 |
|  |  | rs77924615 | A | G | -0.015792 | 0.002464 | 0.197773 | 1.52E-10 | 0.0001 | 41 |
|  |  | rs7812435 | G | T | 0.011015 | 0.002198 | 0.729136 | 5.51E-07 | 0.0001 | 25 |
|  |  | rs7828742 | G | A | -0.02193 | 0.00199 | 0.596853 | 3.40E-28 | 0.0003 | 121 |
|  |  | rs784887 | G | T | -0.01377 | 0.002556 | 0.180672 | 7.30E-08 | 0.0001 | 29 |
|  |  | rs78644250 | G | C | -0.014573 | 0.002651 | 0.18209 | 3.94E-08 | 0.0001 | 30 |
|  |  | rs78649910 | A | T | -0.018331 | 0.003122 | 0.110004 | 4.41E-09 | 0.0001 | 34 |
|  |  | rs7910135 | A | C | 0.01021 | 0.001944 | 0.519138 | 1.53E-07 | 0.0001 | 28 |
|  |  | rs79598313 | T | C | -0.032989 | 0.006425 | 0.023469 | 2.89E-07 | 0.0001 | 26 |
|  |  | rs7981402 | A | G | 0.010448 | 0.002055 | 0.340094 | 3.78E-07 | 0.0001 | 26 |
|  |  | rs80204526 | A | C | -0.048218 | 0.00942 | 0.011119 | 3.14E-07 | 0.0001 | 26 |
|  |  | rs80237449 | G | A | 0.017823 | 0.003395 | 0.090841 | 1.56E-07 | 0.0001 | 28 |
|  |  | rs804280 | A | C | 0.013033 | 0.001978 | 0.581959 | 4.61E-11 | 0.0001 | 43 |
|  |  | rs8063706 | T | A | 0.012968 | 0.002198 | 0.272828 | 3.72E-09 | 0.0001 | 35 |
|  |  | rs8086642 | A | G | -0.009711 | 0.001952 | 0.540154 | 6.67E-07 | 0.0001 | 25 |
|  |  | rs8091117 | A | C | -0.024071 | 0.003943 | 0.065396 | 1.06E-09 | 0.0001 | 37 |
|  |  | rs814573 | T | A | -0.01508 | 0.002642 | 0.183339 | 1.18E-08 | 0.0001 | 33 |
|  |  | rs867772 | G | A | -0.013838 | 0.002091 | 0.681808 | 3.75E-11 | 0.0001 | 44 |
|  |  | rs9423639 | T | C | -0.011997 | 0.002185 | 0.274575 | 4.08E-08 | 0.0001 | 30 |
|  |  | rs942380 | G | A | 0.011434 | 0.001981 | 0.594833 | 8.10E-09 | 0.0001 | 33 |
|  |  | rs9476310 | T | C | 0.011133 | 0.001948 | 0.511302 | 1.13E-08 | 0.0001 | 33 |
|  |  | rs9536961 | G | A | -0.011759 | 0.00207 | 0.346767 | 1.37E-08 | 0.0001 | 32 |
|  |  | rs960596 | T | C | 0.012414 | 0.002076 | 0.339588 | 2.29E-09 | 0.0001 | 36 |
|  |  | rs964184 | C | G | 0.03977 | 0.002858 | 0.863547 | 5.73E-44 | 0.0004 | 194 |
|  |  | rs9668081 | T | C | 0.0116 | 0.001988 | 0.47058 | 5.49E-09 | 0.0001 | 34 |

SNPs with r2 < 0.001 within 10,000 kb windows and P ≤ 5E-08 were used for the main analyses, while SNPs with r2 < 0.001 within 10,000 kb and P ≤ 1E-06 were used for the secondary analyses.

Abbreviations: Cu, Copper; EA, effect allele; EAF, effect allele frequency; OA, other allele; R2, Proportion of the explained variance; Se, Selenium; Std, standard error; Zn, Zinc.

Note: SNPs with missing data had been excluded before the analysis. The pval and MAF are used to select SNPs, and the beta, standard error and MAF are used to calculate the F statistic and the proportion of variance interpreted (R2) to measure the power of the IVs screened and to ensure their close association with exposures. The number of SNPs used in our study ranged from 1 to 164. The F-statistics exceeded 10 for all the IVs indicating that the selected IVs fulfilled the first assumption.

**Table S3.** Main mendelian randomization analyses of micronutrients

| Metabolitis | SNP | heterogeneity test | | | IVW model | OR | 95%CI | P value | Pleio (MR-Egger) |
| --- | --- | --- | --- | --- | --- | --- | --- | --- | --- |
|  |  | MR-Egger.p | IVW.p | |  |  |  |  |  |
| Cu | 2 | - | | 3.24E-01 | FEM | 1.01E+00 | 0.88-1.16 | 8.81E-01 | - |
| Se-Q | 1 | - | | - | FEM | - | - | - | - |
| Se-A | 1 | - | | - | FEM | - | - | - | - |
| Zn | 2 | - | | 7.23E-01 | FEM | 1.23E+00 | 1.07-1.43 | 4.26E-03 | - |
| Vitamin B6 | 1 | - | | - | MRE | - | - | - | - |
| Vitamin B12 | 6 | 1.74E-02 | | 1.66E-02 | FEM | 1.17E+00 | 0.74-1.85 | 5.05E-01 | 5.01E-01 |
| Vitamin C | 11 | 9.84E-02 | | 1.39E-01 | FEM | 9.09E-01 | 0.73-1.13 | 3.88E-01 | 7.88E-01 |
| Vitamin D | 105 | 3.11E-01 | | 3.36E-01 | FEM | 9.78E-01 | 0.81-1.18 | 8.19E-01 | 9.62E-01 |
| Vitamin E | 0 | - | | - | - | - | - | - | - |

SNPs with r2 < 0.001 within 10,000 kb windows and P ≤ 5E-08 were used for the main analyses.

Abbreviations: Cu, Copper; Se-A, Selenium ALSPAC; Se-Q, Selenium QIMR; Zn, Zinc; FEM: Fixed-effect model; MRE: multiplicative random effects.

**Table S4.** Secondary mendelian randomization analyses of micronutrients

| Metabolitis | SNP | heterogeneity test | | | IVW model | OR | 95%CI | P value | Pleio (MR-Egger) |
| --- | --- | --- | --- | --- | --- | --- | --- | --- | --- |
|  |  | Egger.p | IVW.p | |  |  |  |  |  |
| Cu | 2.00E+00 | - | | 3.24E-01 | FEM | 1.01E+00 | 0.88-1.16 | 8.81E-01 | - |
| Se-Q | 3.00E+00 | 9.60E-01 | | 3.25E-01 | FEM | 1.03E+00 | 0.92-1.15 | 5.98E-01 | 3.75E-03 |
| Se-A | 3.00E+00 | 5.78E-01 | | 8.49E-01 | FEM | 9.73E-01 | 0.85-1.12 | 6.90E-01 | 9.18E-01 |
| Zn | 5.00E+00 | 3.46E-01 | | 4.49E-01 | FEM | 1.13E+00 | 1.02-1.27 | 2.52E-02 | 5.96E-01 |
| Vitamin B6 | 4.00E+00 | 1.74E-01 | | 2.57E-01 | FEM | 2.78E+00 | 1.34-5.76 | 6.04E-03 | 6.33E-01 |
| Vitamin B12 | 6.00E+00 | 4.24E-02 | | 6.65E-02 | FEM | 8.66E-01 | 0.63-1.19 | 3.75E-01 | 1.30E+01 |
| Vitamin C | 1.70E+01 | 1.87E-01 | | 2.36E-01 | FEM | 9.19E-01 | 0.75-1.12 | 4.10E-01 | 8.02E-01 |
| Vitamin D | 1.64E+02 | 1.10E-01 | | 1.21E-01 | FEM | 8.48E-02 | 0.71-1.02 | 7.42E-02 | 9.78E-01 |
| Vitamin E | 6.00E+00 | 6.78E+00 | | 2.38E-01 | FEM | 7.95E-01 | 0.43-1.47 | 4.66E-01 | 9.47E-01 |

SNPs with r2 < 0.001 within 10,000 kb and P ≤ 1E-06 were used for the secondary analyses.

Abbreviations: Cu, Copper; Se-A, Selenium ALSPAC; Se-Q, Selenium QIMR; Zn, Zinc; FEM: Fixed-effect model; MRE: multiplicative random effects.

**Table S5**. Inverse-variance weighting Mendelian randomization studies regression results for the leave one SNP out analysis in the Mendelian randomization analyses of micronutrients in the main analysis

|  | SNP | b | se | p |
| --- | --- | --- | --- | --- |
| Vitamin B12 | rs1131603 | -1.85E-01 | 2.86E-01 | 5.17E-01 |
|  | rs12780845 | -3.21E-01 | 1.66E-01 | 5.32E-02 |
|  | rs1801222 | 7.86E-02 | 2.15E-01 | 7.15E-01 |
|  | rs526934 | -1.74E-01 | 3.08E-01 | 5.73E-01 |
|  | rs6458690 | -1.38E-01 | 2.99E-01 | 6.44E-01 |
| Vitamin C | rs10051765 | -8.12E-02 | 1.44E-01 | 5.74E-01 |
|  | rs10136000 | -1.03E-01 | 1.46E-01 | 4.83E-01 |
|  | rs117885456 | -1.79E-01 | 1.14E-01 | 1.16E-01 |
|  | rs13028225 | -1.18E-01 | 1.53E-01 | 4.43E-01 |
|  | rs174547 | -6.81E-02 | 1.36E-01 | 6.17E-01 |
|  | rs2559850 | -7.03E-02 | 1.48E-01 | 6.35E-01 |
|  | rs33972313 | -4.28E-02 | 1.97E-01 | 8.28E-01 |
|  | rs56738967 | -8.90E-02 | 1.47E-01 | 5.44E-01 |
|  | rs6693447 | -1.08E-01 | 1.46E-01 | 4.57E-01 |
|  | rs9895661 | -7.56E-02 | 1.46E-01 | 6.04E-01 |
| Vitamin D | rs1011468 | -1.44E-02 | 9.81E-02 | 8.84E-01 |
|  | rs1047891 | -1.80E-02 | 9.86E-02 | 8.55E-01 |
|  | rs1048328 | -2.33E-02 | 9.89E-02 | 8.14E-01 |
|  | rs10771090 | -2.52E-02 | 9.86E-02 | 7.98E-01 |
|  | rs10859995 | -1.64E-02 | 1.00E-01 | 8.70E-01 |
|  | rs10864726 | -1.75E-02 | 9.84E-02 | 8.59E-01 |
|  | rs10887718 | -2.36E-02 | 9.88E-02 | 8.11E-01 |
|  | rs10908469 | -2.85E-02 | 9.84E-02 | 7.72E-01 |
|  | rs11127048 | -2.63E-02 | 9.88E-02 | 7.90E-01 |
|  | rs111529171 | -3.09E-02 | 9.76E-02 | 7.52E-01 |
|  | rs112285002 | -9.20E-05 | 1.00E-01 | 9.99E-01 |
|  | rs1137844 | -3.11E-02 | 9.77E-02 | 7.51E-01 |
|  | rs11500197 | -1.29E-02 | 9.71E-02 | 8.94E-01 |
|  | rs11542462 | -1.80E-02 | 9.87E-02 | 8.56E-01 |
|  | rs116472025 | -2.38E-02 | 9.88E-02 | 8.10E-01 |
|  | rs11846838 | -8.13E-03 | 9.58E-02 | 9.32E-01 |
|  | rs12123821 | -2.67E-02 | 9.97E-02 | 7.88E-01 |
|  | rs12196316 | -2.17E-02 | 9.88E-02 | 8.26E-01 |
|  | rs12278461 | 3.92E-02 | 1.06E-01 | 7.12E-01 |
|  | rs1229984 | -1.92E-02 | 9.88E-02 | 8.46E-01 |
|  | rs12317268 | -3.01E-02 | 9.79E-02 | 7.58E-01 |
|  | rs12462826 | -2.47E-02 | 9.87E-02 | 8.02E-01 |
|  | rs13022972 | -2.95E-02 | 9.77E-02 | 7.62E-01 |
|  | rs13104531 | -2.54E-02 | 9.87E-02 | 7.97E-01 |
|  | rs13245123 | -2.02E-02 | 9.88E-02 | 8.38E-01 |
|  | rs13284054 | -2.15E-02 | 9.88E-02 | 8.28E-01 |
|  | rs1660818 | -2.33E-02 | 9.88E-02 | 8.14E-01 |
|  | rs1714363 | -2.24E-02 | 9.88E-02 | 8.20E-01 |
|  | rs174418 | -1.14E-02 | 9.86E-02 | 9.08E-01 |
|  | rs17651741 | -1.79E-02 | 9.84E-02 | 8.56E-01 |
|  | rs1800588 | -2.81E-02 | 9.91E-02 | 7.77E-01 |
|  | rs1800775 | -1.53E-02 | 9.86E-02 | 8.76E-01 |
|  | rs187245277 | -2.77E-02 | 9.86E-02 | 7.79E-01 |
|  | rs1950039 | -2.88E-02 | 9.83E-02 | 7.69E-01 |
|  | rs1972994 | -2.72E-02 | 9.87E-02 | 7.83E-01 |
|  | rs2012736 | -2.46E-02 | 9.93E-02 | 8.04E-01 |
|  | rs2037511 | -2.55E-02 | 9.86E-02 | 7.96E-01 |
|  | rs2074735 | -2.62E-02 | 9.86E-02 | 7.90E-01 |
|  | rs2144530 | -2.71E-02 | 9.92E-02 | 7.85E-01 |
|  | rs2229742 | -2.44E-02 | 9.89E-02 | 8.05E-01 |
|  | rs2245133 | -9.40E-03 | 9.73E-02 | 9.23E-01 |
|  | rs2762938 | -2.02E-02 | 9.88E-02 | 8.38E-01 |
|  | rs27774 | -1.89E-02 | 9.86E-02 | 8.48E-01 |
|  | rs28393895 | -4.63E-03 | 1.04E-01 | 9.64E-01 |
|  | rs2847500 | -2.39E-02 | 9.88E-02 | 8.09E-01 |
|  | rs2909218 | -2.24E-02 | 9.89E-02 | 8.21E-01 |
|  | rs2934744 | -2.24E-02 | 9.91E-02 | 8.21E-01 |
|  | rs34726834 | -1.61E-02 | 9.82E-02 | 8.70E-01 |
|  | rs36037728 | -3.39E-02 | 9.79E-02 | 7.29E-01 |
|  | rs373397810 | -2.10E-02 | 9.88E-02 | 8.31E-01 |
|  | rs3750296 | -4.12E-02 | 9.67E-02 | 6.70E-01 |
|  | rs4121823 | -1.93E-02 | 9.87E-02 | 8.45E-01 |
|  | rs4267257 | -2.11E-02 | 9.88E-02 | 8.31E-01 |
|  | rs4364259 | -2.30E-02 | 9.88E-02 | 8.16E-01 |
|  | rs4616820 | -2.25E-02 | 9.88E-02 | 8.20E-01 |
|  | rs4635554 | -1.51E-02 | 9.80E-02 | 8.78E-01 |
|  | rs466360 | -1.54E-02 | 9.80E-02 | 8.75E-01 |
|  | rs523583 | -2.26E-02 | 9.88E-02 | 8.19E-01 |
|  | rs532436 | -1.34E-02 | 9.75E-02 | 8.91E-01 |
|  | rs55886116 | -1.87E-02 | 9.86E-02 | 8.49E-01 |
|  | rs58542926 | -1.17E-02 | 9.81E-02 | 9.05E-01 |
|  | rs6127099 | -1.60E-02 | 9.97E-02 | 8.72E-01 |
|  | rs61747728 | -2.85E-02 | 9.81E-02 | 7.72E-01 |
|  | rs61815559 | -8.57E-03 | 9.91E-02 | 9.31E-01 |
|  | rs6438900 | -1.31E-02 | 9.75E-02 | 8.93E-01 |
|  | rs6698680 | -2.36E-02 | 9.88E-02 | 8.11E-01 |
|  | rs6724965 | -1.89E-02 | 9.87E-02 | 8.48E-01 |
|  | rs6773343 | -1.78E-02 | 9.85E-02 | 8.56E-01 |
|  | rs6954700 | -1.95E-02 | 9.87E-02 | 8.43E-01 |
|  | rs71383766 | -2.30E-02 | 9.88E-02 | 8.16E-01 |
|  | rs7178572 | -1.59E-02 | 9.83E-02 | 8.72E-01 |
|  | rs72632970 | -1.93E-02 | 1.01E-01 | 8.49E-01 |
|  | rs72645665 | -5.58E-02 | 1.03E-01 | 5.89E-01 |
|  | rs72665698 | -3.14E-02 | 9.72E-02 | 7.46E-01 |
|  | rs72897736 | -2.86E-02 | 9.80E-02 | 7.71E-01 |
|  | rs73015021 | -3.66E-02 | 9.65E-02 | 7.05E-01 |
|  | rs7314285 | -1.57E-02 | 9.80E-02 | 8.73E-01 |
|  | rs7439098 | -5.03E-02 | 9.67E-02 | 6.03E-01 |
|  | rs7519574 | -1.70E-02 | 9.85E-02 | 8.63E-01 |
|  | rs7528419 | -2.60E-02 | 9.88E-02 | 7.92E-01 |
|  | rs7569755 | -1.06E-02 | 9.69E-02 | 9.13E-01 |
|  | rs7650253 | -2.85E-02 | 9.82E-02 | 7.72E-01 |
|  | rs7691791 | -2.15E-02 | 9.88E-02 | 8.28E-01 |
|  | rs7718395 | -1.81E-02 | 9.85E-02 | 8.54E-01 |
|  | rs7724488 | -2.07E-02 | 9.88E-02 | 8.34E-01 |
|  | rs77532868 | -1.55E-02 | 9.81E-02 | 8.74E-01 |
|  | rs77924615 | -2.57E-02 | 9.86E-02 | 7.94E-01 |
|  | rs7828742 | -3.31E-02 | 9.85E-02 | 7.37E-01 |
|  | rs78644250 | -2.22E-02 | 9.88E-02 | 8.22E-01 |
|  | rs78649910 | -2.20E-02 | 9.88E-02 | 8.24E-01 |
|  | rs7938266 | -3.98E-02 | 1.03E-01 | 7.00E-01 |
|  | rs804280 | -1.72E-02 | 9.85E-02 | 8.61E-01 |
|  | rs8063706 | -2.09E-02 | 9.88E-02 | 8.33E-01 |
|  | rs8091117 | -2.07E-02 | 9.88E-02 | 8.34E-01 |
|  | rs814573 | -2.34E-02 | 9.88E-02 | 8.13E-01 |
|  | rs867772 | -2.85E-02 | 9.82E-02 | 7.72E-01 |
|  | rs9423639 | -2.56E-02 | 9.85E-02 | 7.95E-01 |
|  | rs942380 | -3.23E-02 | 9.67E-02 | 7.38E-01 |
|  | rs9476310 | -2.60E-02 | 9.85E-02 | 7.92E-01 |
|  | rs9536961 | -2.57E-02 | 9.85E-02 | 7.94E-01 |
|  | rs960596 | -1.81E-02 | 9.86E-02 | 8.54E-01 |
|  | rs964184 | -2.62E-02 | 9.94E-02 | 7.92E-01 |
|  | rs9668081 | -1.85E-02 | 9.86E-02 | 8.51E-01 |

**Table S6**. Inverse-variance weighting Mendelian randomization regression results for the leave one SNP out analysis in the Mendelian randomization analyses of micronutrients in the secondary analysis

|  | SNP | b | se | p | |
| --- | --- | --- | --- | --- | --- |
| Se QIMR | rs10023369 | 5.68E-02 | 5.90E-02 | 3.36E-01 | |
|  | rs4950779 | -3.62E-02 | 7.61E-02 | 6.34E-01 | |
|  | rs7700970 | 4.44E-02 | 1.01E-01 | 6.60E-01 | |
| Se ALSPAC | rs11948804 | -1.44E-02 | 7.80E-02 | 8.54E-01 | |
|  | rs12951643 | -4.43E-02 | 7.74E-02 | 5.67E-01 | |
|  | rs921943 | -2.11E-02 | 1.14E-01 | 8.53E-01 | |
| Zn | rs11232535 | 1.43E-01 | 6.33E-02 | | 2.34E-02 |
|  | rs11763353 | 1.52E-01 | 5.96E-02 | | 1.06E-02 |
|  | rs1532423 | 1.11E-01 | 6.82E-02 | | 1.03E-01 |
|  | rs2120019 | 6.61E-02 | 7.01E-02 | | 3.46E-01 |
|  | rs7148590 | 1.38E-01 | 6.43E-02 | | 3.23E-02 |
| Vitamin B6 | rs12226112 | 9.43E-01 | 5.89E-01 | | 1.09E-01 |
|  | rs155599 | 1.29E+00 | 5.29E-01 | | 1.46E-02 |
|  | rs3772928 | 1.22E+00 | 5.48E-01 | | 2.61E-02 |
|  | rs67450584 | 6.70E-01 | 4.22E-01 | | 1.12E-01 |
| Vitamin B12 | rs1131603 | -1.63E-01 | 1.82E-01 | | 3.69E-01 |
|  | rs12780845 | -2.62E-01 | 1.25E-01 | | 3.64E-02 |
|  | rs1801222 | 2.19E-02 | 1.43E-01 | | 8.78E-01 |
|  | rs2972578 | -1.24E-01 | 1.81E-01 | | 4.92E-01 |
|  | rs4458686 | -1.28E-01 | 1.88E-01 | | 4.94E-01 |
|  | rs526934 | -1.53E-01 | 1.92E-01 | | 4.25E-01 |
|  | rs61960878 | -1.40E-01 | 1.81E-01 | | 4.41E-01 |
|  | rs79324875 | -1.78E-01 | 1.76E-01 | | 3.13E-01 |
| Vitamin C | rs10051765 | -7.17E-02 | 1.18E-01 | | 5.44E-01 |
|  | rs10136000 | -8.97E-02 | 1.19E-01 | | 4.51E-01 |
|  | rs1165189 | -8.66E-02 | 1.19E-01 | | 4.67E-01 |
|  | rs117885456 | -1.54E-01 | 1.05E-01 | | 1.41E-01 |
|  | rs13028225 | -1.01E-01 | 1.24E-01 | | 4.16E-01 |
|  | rs174547 | -6.06E-02 | 1.13E-01 | | 5.92E-01 |
|  | rs2366388 | -6.68E-02 | 1.15E-01 | | 5.60E-01 |
|  | rs2559850 | -6.20E-02 | 1.20E-01 | | 6.06E-01 |
|  | rs2941484 | -6.53E-02 | 1.15E-01 | | 5.70E-01 |
|  | rs33972313 | -3.68E-02 | 1.51E-01 | | 8.08E-01 |
|  | rs56738967 | -7.82E-02 | 1.19E-01 | | 5.12E-01 |
|  | rs6482188 | -1.02E-01 | 1.15E-01 | | 3.72E-01 |
|  | rs6693447 | -9.45E-02 | 1.19E-01 | | 4.27E-01 |
|  | rs676317 | -9.62E-02 | 1.17E-01 | | 4.12E-01 |
|  | rs79234109 | -9.81E-02 | 1.17E-01 | | 4.01E-01 |
|  | rs9895661 | -6.67E-02 | 1.19E-01 | | 5.75E-01 |
| Vitamin E | rs111306778 | -5.24E-01 | 3.46E-01 | 1.29E-01 | |
|  | rs12165526 | -2.81E-01 | 4.48E-01 | 5.31E-01 | |
|  | rs4903544 | -3.75E-02 | 3.78E-01 | 9.21E-01 | |
|  | rs536912 | -2.60E-01 | 4.46E-01 | 5.60E-01 | |
|  | rs6033 | -2.36E-01 | 4.47E-01 | 5.97E-01 | |
|  | rs71385328 | -4.09E-02 | 3.88E-01 | 9.16E-01 | |
| Vitamin D | rs10101205 | -1.69E-01 | 9.81E-02 | 8.51E-02 | |
|  | rs1011468 | -1.58E-01 | 9.82E-02 | 1.07E-01 | |
|  | rs10128681 | -1.94E-01 | 1.03E-01 | 5.83E-02 | |
|  | rs1047891 | -1.61E-01 | 9.84E-02 | 1.01E-01 | |
|  | rs1048328 | -1.66E-01 | 9.86E-02 | 9.13E-02 | |
|  | rs10758321 | -1.58E-01 | 9.77E-02 | 1.06E-01 | |
|  | rs10771090 | -1.68E-01 | 9.83E-02 | 8.77E-02 | |
|  | rs10794655 | -1.56E-01 | 9.70E-02 | 1.09E-01 | |
|  | rs10859995 | -1.64E-01 | 9.98E-02 | 1.01E-01 | |
|  | rs10864726 | -1.61E-01 | 9.83E-02 | 1.02E-01 | |
|  | rs10887718 | -1.67E-01 | 9.85E-02 | 9.09E-02 | |
|  | rs10896045 | -1.72E-01 | 9.75E-02 | 7.78E-02 | |
|  | rs10898241 | -1.62E-01 | 1.03E-01 | 1.14E-01 | |
|  | rs10908469 | -1.71E-01 | 9.82E-02 | 8.14E-02 | |
|  | rs11057274 | -1.62E-01 | 9.84E-02 | 9.87E-02 | |
|  | rs11127048 | -1.69E-01 | 9.85E-02 | 8.56E-02 | |
|  | rs111529171 | -1.73E-01 | 9.77E-02 | 7.62E-02 | |
|  | rs11209952 | -1.65E-01 | 9.85E-02 | 9.41E-02 | |
|  | rs112285002 | -1.50E-01 | 1.00E-01 | 1.34E-01 | |
|  | rs112736716 | -1.58E-01 | 9.79E-02 | 1.05E-01 | |
|  | rs112989220 | -1.65E-01 | 9.85E-02 | 9.47E-02 | |
|  | rs113209890 | -1.60E-01 | 9.92E-02 | 1.08E-01 | |
|  | rs1137844 | -1.73E-01 | 9.78E-02 | 7.60E-02 | |
|  | rs11500197 | -1.57E-01 | 9.75E-02 | 1.09E-01 | |
|  | rs11542462 | -1.61E-01 | 9.85E-02 | 1.01E-01 | |
|  | rs11602347 | -1.72E-01 | 9.74E-02 | 7.70E-02 | |
|  | rs11624558 | -1.66E-01 | 9.84E-02 | 9.22E-02 | |
|  | rs116472025 | -1.67E-01 | 9.85E-02 | 9.05E-02 | |
|  | rs117862422 | -1.59E-01 | 9.79E-02 | 1.05E-01 | |
|  | rs117962481 | -1.60E-01 | 9.82E-02 | 1.03E-01 | |
|  | rs11846838 | -1.52E-01 | 9.67E-02 | 1.16E-01 | |
|  | rs11885466 | -1.69E-01 | 9.81E-02 | 8.53E-02 | |
|  | rs12123821 | -1.72E-01 | 9.92E-02 | 8.35E-02 | |
|  | rs1214761 | -1.71E-01 | 9.78E-02 | 8.09E-02 | |
|  | rs12196316 | -1.65E-01 | 9.85E-02 | 9.46E-02 | |
|  | rs1229984 | -1.63E-01 | 9.86E-02 | 9.90E-02 | |
|  | rs12317268 | -1.73E-01 | 9.79E-02 | 7.79E-02 | |
|  | rs12462826 | -1.67E-01 | 9.84E-02 | 8.87E-02 | |
|  | rs12554549 | -1.66E-01 | 9.84E-02 | 9.09E-02 | |
|  | rs12964203 | -1.65E-01 | 9.85E-02 | 9.35E-02 | |
|  | rs13022972 | -1.72E-01 | 9.78E-02 | 7.87E-02 | |
|  | rs13030535 | -1.66E-01 | 9.84E-02 | 9.22E-02 | |
|  | rs13123929 | -1.60E-01 | 9.81E-02 | 1.03E-01 | |
|  | rs13137545 | -1.55E-01 | 9.78E-02 | 1.12E-01 | |
|  | rs13245123 | -1.63E-01 | 9.85E-02 | 9.74E-02 | |
|  | rs13278404 | -1.57E-01 | 9.76E-02 | 1.07E-01 | |
|  | rs13284054 | -1.64E-01 | 9.85E-02 | 9.50E-02 | |
|  | rs1484423 | -1.68E-01 | 9.82E-02 | 8.65E-02 | |
|  | rs1542926 | -1.60E-01 | 9.82E-02 | 1.02E-01 | |
|  | rs1714363 | -1.65E-01 | 9.85E-02 | 9.32E-02 | |
|  | rs17161467 | -1.46E-01 | 9.72E-02 | 1.33E-01 | |
|  | rs174418 | -1.56E-01 | 9.85E-02 | 1.13E-01 | |
|  | rs17616063 | -1.63E-01 | 9.84E-02 | 9.68E-02 | |
|  | rs17651741 | -1.61E-01 | 9.83E-02 | 1.01E-01 | |
|  | rs1790337 | -1.56E-01 | 9.95E-02 | 1.17E-01 | |
|  | rs1800588 | -1.72E-01 | 9.88E-02 | 8.19E-02 | |
|  | rs1800775 | -1.59E-01 | 9.85E-02 | 1.06E-01 | |
|  | rs183717655 | -1.65E-01 | 9.85E-02 | 9.38E-02 | |
|  | rs1843096 | -1.70E-01 | 9.79E-02 | 8.24E-02 | |
|  | rs1883711 | -1.62E-01 | 9.84E-02 | 1.00E-01 | |
|  | rs1909585 | -1.64E-01 | 9.85E-02 | 9.62E-02 | |
|  | rs1950039 | -1.71E-01 | 9.82E-02 | 8.06E-02 | |
|  | rs1972994 | -1.70E-01 | 9.84E-02 | 8.39E-02 | |
|  | rs2012736 | -1.69E-01 | 9.89E-02 | 8.80E-02 | |
|  | rs2037511 | -1.68E-01 | 9.83E-02 | 8.71E-02 | |
|  | rs2074735 | -1.69E-01 | 9.83E-02 | 8.57E-02 | |
|  | rs2144530 | -1.71E-01 | 9.88E-02 | 8.36E-02 | |
|  | rs2157829 | -1.59E-01 | 9.79E-02 | 1.05E-01 | |
|  | rs2229742 | -1.67E-01 | 9.85E-02 | 8.93E-02 | |
|  | rs2245133 | -1.54E-01 | 9.78E-02 | 1.16E-01 | |
|  | rs2334097 | -1.70E-01 | 9.79E-02 | 8.20E-02 | |
|  | rs2544725 | -1.64E-01 | 9.85E-02 | 9.57E-02 | |
|  | rs2647463 | -1.68E-01 | 9.83E-02 | 8.81E-02 | |
|  | rs2762938 | -1.63E-01 | 9.85E-02 | 9.72E-02 | |
|  | rs27774 | -1.62E-01 | 9.84E-02 | 9.96E-02 | |
|  | rs28437159 | -1.79E-01 | 9.98E-02 | 7.36E-02 | |
|  | rs2847500 | -1.67E-01 | 9.85E-02 | 9.02E-02 | |
|  | rs28798705 | -1.61E-01 | 9.83E-02 | 1.01E-01 | |
|  | rs2909218 | -1.65E-01 | 9.85E-02 | 9.31E-02 | |
|  | rs2934744 | -1.66E-01 | 9.88E-02 | 9.24E-02 | |
|  | rs34560261 | -1.69E-01 | 9.82E-02 | 8.55E-02 | |
|  | rs34726834 | -1.60E-01 | 9.82E-02 | 1.04E-01 | |
|  | rs35535415 | -1.66E-01 | 9.84E-02 | 9.24E-02 | |
|  | rs35846253 | -1.41E-01 | 1.00E-01 | 1.59E-01 | |
|  | rs373397810 | -1.64E-01 | 9.85E-02 | 9.57E-02 | |
|  | rs3750296 | -1.83E-01 | 9.72E-02 | 5.90E-02 | |
|  | rs4121823 | -1.62E-01 | 9.84E-02 | 9.90E-02 | |
|  | rs4267257 | -1.64E-01 | 9.85E-02 | 9.57E-02 | |
|  | rs4364259 | -1.66E-01 | 9.85E-02 | 9.21E-02 | |
|  | rs4553272 | -1.69E-01 | 9.82E-02 | 8.51E-02 | |
|  | rs4603973 | -1.57E-01 | 9.77E-02 | 1.08E-01 | |
|  | rs4616820 | -1.65E-01 | 9.85E-02 | 9.30E-02 | |
|  | rs4635554 | -1.59E-01 | 9.81E-02 | 1.06E-01 | |
|  | rs466360 | -1.59E-01 | 9.80E-02 | 1.05E-01 | |
|  | rs4738679 | -1.62E-01 | 9.83E-02 | 9.99E-02 | |
|  | rs4788475 | -1.65E-01 | 9.85E-02 | 9.43E-02 | |
|  | rs499974 | -1.64E-01 | 9.85E-02 | 9.62E-02 | |
|  | rs512083 | -1.68E-01 | 9.82E-02 | 8.62E-02 | |
|  | rs532436 | -1.57E-01 | 9.78E-02 | 1.08E-01 | |
|  | rs55886116 | -1.62E-01 | 9.84E-02 | 9.99E-02 | |
|  | rs56185965 | -1.58E-01 | 9.77E-02 | 1.06E-01 | |
|  | rs56257628 | -1.59E-01 | 9.80E-02 | 1.04E-01 | |
|  | rs58542926 | -1.56E-01 | 9.82E-02 | 1.13E-01 | |
|  | rs59789656 | -1.75E-01 | 9.69E-02 | 7.17E-02 | |
|  | rs6127099 | -1.62E-01 | 9.94E-02 | 1.03E-01 | |
|  | rs61747728 | -1.71E-01 | 9.80E-02 | 8.11E-02 | |
|  | rs61815559 | -1.55E-01 | 9.90E-02 | 1.19E-01 | |
|  | rs61887421 | -1.65E-01 | 9.85E-02 | 9.31E-02 | |
|  | rs6438900 | -1.57E-01 | 9.78E-02 | 1.09E-01 | |
|  | rs6698680 | -1.66E-01 | 9.85E-02 | 9.09E-02 | |
|  | rs6724965 | -1.62E-01 | 9.85E-02 | 9.96E-02 | |
|  | rs6773343 | -1.61E-01 | 9.83E-02 | 1.01E-01 | |
|  | rs6858160 | -1.71E-01 | 1.03E-01 | 9.80E-02 | |
|  | rs6954700 | -1.63E-01 | 9.84E-02 | 9.86E-02 | |
|  | rs71383766 | -1.66E-01 | 9.85E-02 | 9.21E-02 | |
|  | rs7178572 | -1.59E-01 | 9.83E-02 | 1.05E-01 | |
|  | rs7204844 | -1.64E-01 | 9.84E-02 | 9.67E-02 | |
|  | rs72631431 | -1.63E-01 | 9.84E-02 | 9.85E-02 | |
|  | rs72632970 | -1.69E-01 | 1.01E-01 | 9.38E-02 | |
|  | rs72665698 | -1.74E-01 | 9.74E-02 | 7.46E-02 | |
|  | rs72680101 | -1.64E-01 | 9.85E-02 | 9.65E-02 | |
|  | rs72897736 | -1.71E-01 | 9.80E-02 | 8.08E-02 | |
|  | rs73015021 | -1.79E-01 | 9.71E-02 | 6.55E-02 | |
|  | rs73030358 | -1.63E-01 | 9.84E-02 | 9.74E-02 | |
|  | rs7314285 | -1.59E-01 | 9.81E-02 | 1.05E-01 | |
|  | rs745797 | -1.61E-01 | 9.83E-02 | 1.01E-01 | |
|  | rs74797364 | -1.70E-01 | 9.83E-02 | 8.34E-02 | |
|  | rs7519574 | -1.60E-01 | 9.84E-02 | 1.03E-01 | |
|  | rs7528419 | -1.69E-01 | 9.85E-02 | 8.61E-02 | |
|  | rs7569755 | -1.54E-01 | 9.74E-02 | 1.13E-01 | |
|  | rs75936148 | -1.69E-01 | 9.82E-02 | 8.60E-02 | |
|  | rs7650253 | -1.71E-01 | 9.81E-02 | 8.12E-02 | |
|  | rs7691791 | -1.65E-01 | 9.85E-02 | 9.48E-02 | |
|  | rs7718395 | -1.61E-01 | 9.84E-02 | 1.01E-01 | |
|  | rs77194050 | -1.67E-01 | 9.84E-02 | 9.00E-02 | |
|  | rs7724488 | -1.64E-01 | 9.85E-02 | 9.65E-02 | |
|  | rs77532868 | -1.59E-01 | 9.81E-02 | 1.05E-01 | |
|  | rs77924615 | -1.68E-01 | 9.83E-02 | 8.67E-02 | |
|  | rs7812435 | -1.67E-01 | 9.83E-02 | 8.89E-02 | |
|  | rs7828742 | -1.76E-01 | 9.83E-02 | 7.32E-02 | |
|  | rs784887 | -1.57E-01 | 9.77E-02 | 1.07E-01 | |
|  | rs78644250 | -1.65E-01 | 9.85E-02 | 9.36E-02 | |
|  | rs78649910 | -1.65E-01 | 9.85E-02 | 9.39E-02 | |
|  | rs7910135 | -1.71E-01 | 9.78E-02 | 8.03E-02 | |
|  | rs79598313 | -1.65E-01 | 9.85E-02 | 9.48E-02 | |
|  | rs7981402 | -1.61E-01 | 9.82E-02 | 1.02E-01 | |
|  | rs80204526 | -1.56E-01 | 9.72E-02 | 1.09E-01 | |
|  | rs80237449 | -1.61E-01 | 9.82E-02 | 1.02E-01 | |
|  | rs804280 | -1.61E-01 | 9.84E-02 | 1.03E-01 | |
|  | rs8063706 | -1.64E-01 | 9.85E-02 | 9.60E-02 | |
|  | rs8086642 | -1.60E-01 | 9.81E-02 | 1.02E-01 | |
|  | rs8091117 | -1.64E-01 | 9.85E-02 | 9.64E-02 | |
|  | rs814573 | -1.66E-01 | 9.85E-02 | 9.13E-02 | |
|  | rs867772 | -1.71E-01 | 9.81E-02 | 8.11E-02 | |
|  | rs9423639 | -1.68E-01 | 9.83E-02 | 8.68E-02 | |
|  | rs942380 | -1.75E-01 | 9.71E-02 | 7.24E-02 | |
|  | rs9476310 | -1.69E-01 | 9.83E-02 | 8.61E-02 | |
|  | rs9536961 | -1.68E-01 | 9.83E-02 | 8.67E-02 | |
|  | rs960596 | -1.61E-01 | 9.84E-02 | 1.01E-01 | |
|  | rs964184 | -1.71E-01 | 9.90E-02 | 8.50E-02 | |
|  | rs9668081 | -1.62E-01 | 9.84E-02 | 1.00E-01 | |

**Table S7**. Multivariable Mendelian randomization result of micronutrients

| exposure | Data source | nsnp | OR | OR 95%CI | pval | F-statistic | pleio test | Heterogeneity test |
| --- | --- | --- | --- | --- | --- | --- | --- | --- |
| Zinc | ieu-a-1079 | 1 | 0.98 | 0.90-1.08 | 0.72 | 1.33 | 0.37 | 0.38 |
| Mean corpuscular volume | ukb-d-30040_irnt | 133 | 1.08 | 0.95-1.23 | 0.23 | 24.09 |  |  |
| Mean corpuscular haemoglobin concentration | ukb-d-30060_irnt | 28 | 0.94 | 0.67-1.31 | 0.71 | 12.07 |  |  |
| Reticulocyte count | ukb-d-30250_irnt | 94 | 1.09 | 0.91-1.30 | 0.37 | 24.28 |  |  |

**Table S8.** Reverse Mendelian randomization results of zinc and vitamin B6

| Metabolitis | SNP | heterogeneity test | | IVW model | OR | 95%CI | P value | Pleio (MR-Egger) |
| --- | --- | --- | --- | --- | --- | --- | --- | --- |
|  |  | Egger.p | IVW.p |  |  |  |  |  |
| zinc | 4 | 0.33 | 0.25 | FEM | 0.92 | 0.69-1.23 | 0.57 | 0.32 |
| Vitamin B6 | 18 | 0.43 | 0.50 | FEM | 1.00 | 0.97-1.03 | 0.96 | 0.97 |

Abbreviations: Zn, Zinc; FEM: Fixed-effect model; MRE: multiplicative random effects.

**Table S9**. Data source information

| Exposure/outcome | Population ancestry | Participants included in  the analysis | Data information |
| --- | --- | --- | --- |
| Cu | European | 2603 | This analysis is based on the results for 2603 adults with phenotype and genotype data who participated in one or both of two studies run from the Queensland Institute of Medical Research. Participants completed a postal questionnaire in 1989 and a telephone interview in 1993–1994, and provided a blood sample in 1993–1996.  The Avon Longitudinal Study of Parents and Children(ALSPAC), also known as Children of the Nineties, was designed to understand the ways in which the physical and social environments interact over time with genetic inheritance to affect health, behavior and development in infancy, childhood and then into adulthood. Eligible women were those who were pregnant, resident in the study area and had an expected date of delivery between 1 April 991 and 31 December 1992. |
| Se | European | 2603(QIMR) |  |
|  | European | 8340(ALSPAC) |  |
| Zn | European | 2603 |  |
| Vitamin B6 | European | 64979 | Output from GWAS pipeline using Phesant derived variables from UKBiobank. |
| Vitamin B12 | European | 72824 | This analysis obtained genotype information on 94,474 BioVU individuals of different ancestral and racial backgrounds genotyped on the Illumina MEGAEX array from Vanderbilt University Medical Center. After cleaning, they got 72824 72,824 individuals of European genetic ancestry. |
| Vitamin C | European | 52018 | Meta-analysis of results from Fenland (n=10771),  EPIC-Norfolk (n=16756),  InterAct (n=16841),  EPIC-CVD (n=7650)  Total N: 52018 |
| Vitamin D | European | 440414 | Data on 25OHD level (in nmol/L) measured using the Diasorin assay were available from 465,415 samples, representing 449,978 UK Biobank participants. However, they chose to redefine a White British sample (n=440,414) using genetic information only. |
| Vitamin E | European | 64979 | Output from GWAS pipeline using Phesant derived variables from UKBiobank |
